# Anion exchange beads for PFAS capture using a polymerization-induced microphase separation approach

**DOI:** 10.1039/d6lp00075d

**Published:** 2026-06-17

**Authors:** Ali Arshad, Jongho Back, Katharine A. Faber, William A. Arnold, Philippe Bühlmann, Marc A. Hillmyer

**Affiliations:** a Department of Chemistry, University of Minnesota 207 Pleasant St. SE Minneapolis MN 55455-0431 USA hillmyer@umn.edu buhlmann@umn.edu; b Department of Civil, Environmental and Geo-Engineering, University of Minnesota 500 Pillsbury Dr. SE Minneapolis MN 55455-0431 USA arnol032@umn.edu

## Abstract

Per- and poly-fluoroalkyl substances (PFAS) are contained in various consumer products that include nonstick coatings, packaging materials, cosmetics, and firefighting foams due to their combined hydrophobic and oleophobic properties and chemical and thermal stability. These properties also result in human toxicity and have led to their accumulation in the environment. Several methods are being used to remove PFAS contaminants from the environment, and one common technique involves removal through ion exchange. Polymerization-induced microphase separation (PIMS) enables the synthesis of PFAS-capturing anion exchange beads featuring co-continuous morphology, tunable domain spacing, and high surface accessibility within a mechanically robust crosslinked network. Beads were synthesized using a poly(ε-caprolactone)-*b*-poly(4-vinylbenzyl chloride)-based macro chain transfer agent, styrene, and divinylbenzene. Anion exchange beads were obtained by etching the poly(ε-caprolactone) component and quaternizing the poly(4-vinylbenzyl chloride), and their ion exchange capacity was measured to be 1.00 ± 0.05 mmol g^−1^. The rates of PFAS removal were evaluated using pseudo-second-order kinetic analysis for both short-chain (trifluoroacetic acid [TFA] and perfluorobutanoic acid [PFBA]) and long-chain (perfluorooctanoic acid [PFOA]) PFAS. The initial sorption rates of TFA and PFBA were 2.9 and 2.3 times higher, respectively, in quaternized beads (PB-Q) compared to Amberlite IRA 900 whole resin. In contrast, PFOA exhibited a 1.7 times higher initial sorption rate to IRA 900 whole resin than to PB-Q. Langmuir isotherm analysis indicated significantly stronger affinities of all PFAS for PB-Q than IRA 900, even though the IRA 900 had greater capacity, suggesting that PB-Q is more effective for removing PFAS at low concentrations. Treating the PFAS loaded PB-Q beads with a 1 : 1 v/v mixture of methanol and 1 M NaCl_(aq)_ resulted in 100% PFAS desorption.

## Abstract 2

Polymerization-induced microphase separation (PIMS) was used to synthesize PFAS-capturing anion exchange beads that showed favorable kinetics and capacities compared to a commercial resin.

## Graphical abstract

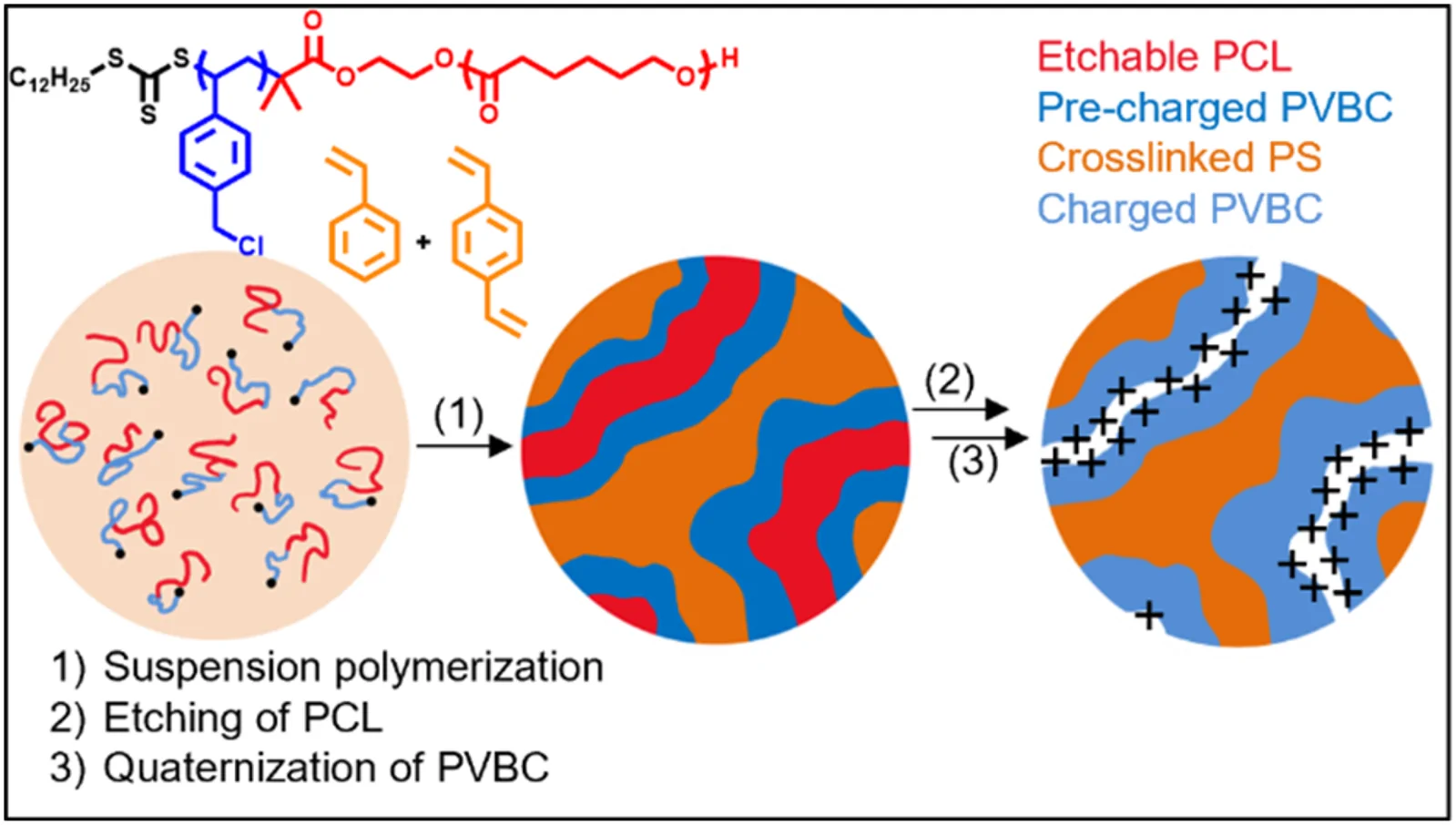

## 1. Introduction

Per- and poly-fluoroalkyl substances (PFAS) are anthropogenic chemicals characterized by a highly fluorinated carbon chain and a terminal functional group.^1,2^ Two main classes of PFAS have either carboxylic acid end groups, known as per- and poly-fluorocarboxylic acids (PFCAs), or sulfonic acid end groups, known as per- and poly-fluoroalkane sulfonic acids (PFSAs). PFCAs with <8 carbon atoms and PFSAs with <6 carbon atoms are known as short-chain PFAS, and those with ≥8 and ≥6 carbon atoms, respectively, are considered long-chain.^3^ PFAS have unique properties due to their combined hydrophobic and oleophobic nature, which makes them suitable for use in various consumer products, including stain-resistant clothing, aqueous firefighting foams, nonstick pans, cosmetic products, and food packaging.^4^ The high chemical and thermal stability of the C–F bonds in PFAS, however, causes them to bioaccumulate and persist in our air, water, and soil.^5–8^ Moreover, PFAS are linked to various health-related problems, including carcinogenicity as well as developmental, genetic, hepatologic, reproductive, immunological, cytologic, neurologic, and hormonal toxicity.^9,10^ Therefore, the U.S. Environmental Protection Agency has established limits on allowable levels of a subset of PFAS in drinking water.^11,12^

Sorption using solid-state media is a prevalent and practical method for capturing PFAS from water. Sorption mechanisms are complex due to the amphiphilicity of many PFAS. On one hand, the hydrophilic head groups of PFCAs and PFSAs are deprotonated at neutral pH because of their low p*K*_a_ values. On the other hand, PFAS also have a hydrophobic and oleophobic tail, the size of which dictates the extent of van der Waals interactions.^13,14^ Adsorption of PFAS often relies on a combination of chemical, electrostatic, hydrogen bond, and van der Waals interactions.^15–17^ Various materials, including granulated/powdered activated carbon, ion exchange resins, granulated cyclodextrin-based resins, metal–organic frameworks, inorganic zeolites, and mesoporous organosilica, have been proposed and used to remove PFAS from contaminated aqueous systems.^18–21^ In particular, anion exchange resins are among the promising adsorbents for PFCA and PFSA removal due to the strong electrostatic interaction between the fixed cationic functional groups of these resins and the anionic head groups of PFCAs and PFSAs. These ionic interactions are often complemented by hydrophobic interactions involving their fluorinated tails and the resin matrix.

Anion exchange resins are typically styrene or acrylate-based polymer networks, generally manufactured through conventional suspension polymerization methods.^22,23^ Precision control of pore sizes, which is critical for tuning PFAS sorption behavior, can be challenging in these classes of materials.^24,25^ Most of these materials require multistep syntheses and dilute solution conditions that hinder scalability. Polymerization-induced microphase separation (PIMS), however, addresses these limitations by combining polymerization and formation of nanostructure morphology in a single step. This approach yields co-continuous morphology with controlled domain sizes and accessible surface areas within a mechanically robust crosslinked network.^26^ Using suspension polymerization, we generate uniform beads of material suitable for packed-bed applications. Architectural precision, permanent-charge functionality, and scalability made PIMS an attractive option for synthesizing strong-base anion-exchange beads for PFAS removal. PIMS utilizes a macromolecular chain transfer agent (macro-CTA) that is extended through reversible addition–fragmentation chain transfer (RAFT) polymerization of monomers and crosslinkers. During polymerization, incompatibility between macro-CTA and the resultant cross-linked network generates phase-separated domains, resulting in a co-continuous morphology.^27^ This technique has been used to prepare materials for various applications, including polymer electrolytes, mesoporous materials, separation membranes, and inks for 3D printing.^28–32^ Anion exchange resin with a mesoporous structure has been successfully prepared using a PIMS approach in a suspension polymerization method, and those materials were effective for the sorption of an anionic dye.^33^

Here, we report on the use of PIMS to synthesize anion exchange beads designed for PFAS removal. A diblock macro-CTA was synthesized using ring-opening polymerization (ROP) of ε-caprolactone (CL), followed by RAFT polymerization of 4-vinylbenzyl chloride (VBC). The macro-CTA diblock was chain-extended with styrene and divinylbenzene using suspension RAFT polymerization to obtain beads as we described previously.^33^ Poly(ε-caprolactone) (PCL) and poly(4-vinylbenzyl chloride) (PVBC) blocks were etched and quaternized, respectively, to obtain the anion exchange beads. PFAS sorption was tested using three different PFAS: trifluoroacetic acid (TFA), perfluorobutanoic acid (PFBA), and perfluorooctanoic acid (PFOA). IRA 900, a commonly utilized commercial anion exchange resin, was used as comparative control because of its structural similarity to our synthesized material. The kinetics and isotherms of sorption were measured, and data were fit with pseudo-second-order, intraparticle diffusion, Langmuir, and Freundlich models. Finally, a successful desorption of PFAS from the anion exchange beads was also demonstrated. This study offers a new synthetic route to making PFAS-sorbing ion exchange materials. This synthesis method is also applicable to other materials with specific ion exchange capacities and ion exchange kinetics.

## 2. Materials and methods

### 2.1. Materials

All the chemicals were purchased commercially and used as received unless mentioned otherwise. ε-Caprolactone (TCI) was distilled under vacuum from calcium hydride (Sigma-Aldrich). Styrene (Sigma-Aldrich), 4-vinylbenzyl chloride (VBC; 90%, Sigma-Aldrich), divinylbenzene (technical grade, Sigma-Aldrich), and ethylene glycol (Fisher Scientific) were passed through a short basic alumina (Sigma-Aldrich) column before use. Poly(vinyl alcohol) (PVA, 15–23 kg mol^−1^, 87–89% hydrolyzed) was purchased from Sigma-Aldrich. Diphenyl phosphate (DPP, Sigma-Aldrich), 4-dimethylamino pyridine (DMAP, Sigma-Aldrich), diisopropylcarbodiimide (DIC, Oakland Chemical), azobisisobutyronitrile (AIBN, Sigma-Aldrich), and lauroyl peroxide (Sigma-Aldrich) were used without further purification. For etching and quaternization, trifluoroacetic acid (TFA, 98%, Sigma-Aldrich) and trimethylamine (TMA, 45% in H_2_O, Sigma-Aldrich) were used as received. Deionized water was purified using a Milli-Q PLUS reagent-grade water filtration system to a resistivity of 18.2 MΩ cm (Millipore, USA) and was used in conductometric titrations. DDMAT-OH, PCL-CTA, and PCL-*b*-PVBC-CTA were synthesized according to the experimental protocols presented in the SI (NMR spectra in Fig. S1–S4). For PFAS sorption experiments, TFA (98%, Sigma-Aldrich) and perfluorooctanoic acid (PFOA, 95%, Sigma-Aldrich) were used in their acidic forms. Perfluorobutanoic acid (PFBA) was used in its salt form, sodium heptafluorobutyrate (98%, Oakwood Chemical). Deionized water was used to make PFAS solutions in batch sorption and isotherm experiments. Commercially available Amberlite IRA 900 (Cl^−^ form, Sigma-Aldrich) anion exchange beads, in both whole and crushed form, were used as a comparison in PFAS sorption experiments. To obtain crushed IRA 900, whole beads were crushed using a porcelain mortar and pestle and passed through steel sieves with mesh size 180 (90 µm) and 300 (50 µm). IRA 900 is a macroporous, strong base, Type I anion exchange resin with wide industrial demineralization and organic scavenging applications.^34^ It is a copolymer of styrene and divinylbenzene, with trimethylammonium methyl (–CH_2_–N^+^(CH_3_)_3_) functional groups.

### 2.2. Instrumentation

Proton nuclear magnetic resonance (^1^H-NMR) spectra were collected in deuterated chloroform (CDCl_3_) using a 400 MHz Bruker Avance III HD spectrometer. Fluorine-NMR (^19^F-NMR) spectra were collected on a 600 MHz Bruker Avance Neo spectrometer equipped with a 5 mm triple resonance cryoprobe. All NMR spectra were processed and analyzed using MestReNova software. Size exclusion chromatography (SEC) chromatograms in tetrahydrofuran (THF, Sigma-Aldrich) were collected using a Wyatt Waters SEC spectrometer equipped with a Wyatt Dawn multi-angle light scattering detector and a Wyatt Optilab differential refractive index detector. Attenuated total reflection–Fourier transform infrared (ATR-FTIR) spectra were obtained using an Agilent Cary 630 FTIR spectrometer. Beads were crushed to a fine powder using a mortar and a pestle before collecting FTIR spectra. Scanning electron microscopy (SEM) images were collected using a Hitachi SU8230 field emission gun scanning electron microscope after coating the beads with 2 nm platinum. An AmScope digital microscope was used to capture bead images and measure size distribution using ImageJ software. Nitrogen sorption isotherms were obtained with a Quantachrome autosorb iQ gas sorption analyzer using a 9 mm stem with a bulb and 70–90 mg beads per sample. All the samples were outgassed at room temperature for 24 h before data collection. The data was processed in ASiQwin software using the Brunauer–Emmett–Teller (BET) model for surface area and the quenched solid density functional theory (QSDFT) model for pore size distribution.

Thermogravimetric analysis (TGA) was performed using a TA Instrument TGA Q500 under a nitrogen atmosphere with a heating rate of 5 °C min^−1^. Small-angle X-ray scattering (SAXS) data were collected from the DND-CAT 5ID-D beamline at the Advanced Photon Source (Argonne, IL) using a photon wavelength of *λ* = 0.7293 Å with a Rayonix MX170-HS detector and a 0.2 s exposure time. Beads were filled in Tzero DSC pans for characterization, and the pan (blank) SAXS data was subtracted from the data. For ion exchange capacity experiments, conductivity and pH were measured using a Hanna Instruments multiparameter benchtop meter – HI2550 equipped with a HI76310 conductivity probe, HI7662 temperature probe, and Cole Palmer pH electrode.

### 2.3. Experimental protocols

#### 2.3.1. Synthesis of the PCL-*b*-PVBC containing beads (PB-S)

To synthesize beads through suspension RAFT polymerization, a 500 mL solution of 0.5 M NaCl_(aq)_ and 2.2 g of PVA were added to a ChemGlass reactor vessel (CG-1920-01). The reactor lid (CG-1940-01) was fitted to the reactor body with a three-blade glass stirring shaft (CG-2079-A-01) mounted to a Heidolph overhead stirrer. The reactor vessel was submerged in an oil bath, and the solution temperature was increased to 70 °C while stirring at 300 rpm to ensure the complete dissolution of PVA. The aqueous solution was then cooled to room temperature and purged with argon for 20 min. Meanwhile, 6 g of PCL-*b*-PVBC-CTA was dissolved in 14 g of styrene : divinylbenzene (2 : 1 molar ratio) in a glass vial, and 44 mg lauroyl peroxide and 2 mL toluene were also added to the vial. Lauroyl peroxide was used as a RAFT polymerization initiator for the suspension polymerization, and PVA was used as a surfactant. The solution was degassed by bubbling argon through it for 10 min. The organic mixture was added to the aqueous solution dropwise using a syringe under constant stirring at 600 rpm using an overhead mechanical stirrer. Then, the temperature of the reaction suspension was increased to 90 °C, and stirring was continued at a constant rate of 600 rpm for 20 h. The reaction suspension was cooled to room temperature, and yellow beads were recovered through vacuum filtration, followed by several washings with water and methanol (MeOH). The recovered beads were dried at 50 °C in a vacuum oven overnight.

#### 2.3.2. Etching of poly(ε-caprolactone) (PCL) block

The PCL block was etched to introduce mesopores in the beads using both alkaline and acidic conditions. For alkaline etching of PCL, 500 mg as-synthesized PCL-*b*-PVBC-*b*-PSDVB beads (PB-S) were stirred (100 rpm) in a 1 : 1 (v/v) blend of MeOH and 3 M aqueous NaOH at 45 °C for 6 d, followed by washing with methanol and drying in the vacuum oven at 50 °C overnight. Acidic etching of PCL was performed by modifying a reported method for the selective etching of poly(lactic acid) (PLA).^35^ PB-S beads (5 g) were stirred (100 rpm) in a 1 : 1 blend (v/v) of TFA and MeOH at 45 °C for 6 h. After 6 h, the beads were transferred into methanol and stirred (200 rpm) for another 6 h to remove the etching by-products, followed by vacuum filtration and oven drying at 50 °C under reduced pressure.

#### 2.3.3. Quaternization of the etched microbeads

The PVBC block was quaternized using TMA to introduce positive charges on the pore walls. 3 g of the etched beads (PB-E) were stirred (100 rpm) in 200 mL TMA at room temperature for 24 h. Then, the beads were filtered and washed with water, followed by air-drying in the fume hood to remove any traces of TMA.

#### 2.3.4. Ion exchange capacity (IEC) measurements

For IEC measurements, a modified conductometric titration method was used.^36^ The quaternized beads (PB-Q) were soaked in 1 M NaCl_(aq)_ solution, followed by sonication at room temperature for 1 h. The beads were removed from the NaCl solution and washed with water before drying them in the vacuum oven at 50 °C overnight. Dried beads (10–20 mg) were added to 2 mL of 100 mM NaNO_3(aq)_ solution and 2 mL of deionized water, and the mixture was sonicated for 1 h to exchange Cl^−^ with NO_3_^−^. After the anion exchange, 170 mL of deionized water was added, and the suspension was stirred at 1000 rpm for 5 min. Titration was conducted by recording the conductivity of the suspension after each addition of a 0.1 mL aliquot of 10 mM AgNO_3(aq)_. Before each conductivity measurement, the suspension was stirred at 1000 rpm for 1 min. Titrations were conducted in triplicate.

The ion exchange capacity was also measured through pH titrations. PB-Q was added to a 20 mL glass vial, covered with glass wool, and 20 mL 1 M NaOH_(aq)_ was added. After 3 d, the NaOH_(aq)_ was replaced with a new batch of alkaline solution. After another three days, NaOH_(aq)_ was removed, and the beads were washed with several portions of water, until the pH of the water was neutral. Then, the beads were dried in a vacuum oven at 50 °C, followed by addition of 20 mL 1 M NaCl_(aq)_ to the vial and letting the system equilibrate. After 5 d, the NaCl_(aq)_ was removed from the vial and titrated against 0.01 N HCl_(aq)_.^37^

#### 2.3.5. PFAS sorption kinetics (batch experiment)

Individual 2 mM solutions of TFA, PFBA, or PFOA in 100 mM phosphate buffer (pH = 7) were used for the batch kinetic experiments to evaluate the PFAS sorption capability of the beads (PB-S, PB-E, PB-Q). For all experiments, PB-Q and IRA 900 were not dried, but the kinetics data were corrected to the dry mass of the beads. For this correction, the dry mass of the beads was measured using TGA and used in all calculations instead of total mass of the beads used. IRA 900 whole resin was used to determine the sorption kinetics of TFA, PFBA, and PFOA, and crushed IRA 900 was also used to determine the batch sorption kinetics of PFBA and PFOA.

Twenty glass vials, each containing 10 mg beads and 16 mL PFAS solution, were prepared for each PFAS-bead type combination. The vials were placed on an orbital shaker at 140 rpm at room temperature. At each time-point, two vials were removed from the orbital shaker to provide duplicate measurements. PFAS solution was collected using a PVDF syringe filter (0.22 µm) to remove beads. PFAS quantification was performed using ^19^F-NMR spectroscopy; details of this analysis are given in section 2.3.8.

Kinetic data were fit to evaluate the sorption kinetics using pseudo-first order (eqn (1)) and pseudo-second order (eqn (2)) kinetic models.

1 *q*_*t*_ = *q*_e_(1 − e^−*k*_1_*t*^)

2
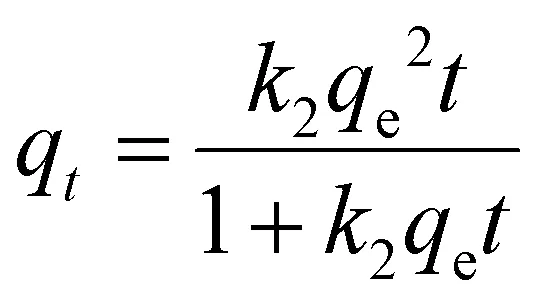
where *q*_*t*_ (mmol g^−1^) and *q*_e_ (mmol g^−1^) are PFAS sorbed onto the dry adsorbent (per unit mass) at time *t* (h) and at equilibrium, respectively. The term *k*_1_ (h^−1^) is the pseudo-first order rate constant, and *k*_2_ (g mmol^−1^ h^−1^) is the pseudo-second order rate constant.^38^ The parameters *q*_e_ and *k*_1_ for pseudo-first order kinetics and *q*_e_ and *k*_2_ for pseudo-second order kinetics were calculated by fitting experimental data to eqn (1) and (2), respectively. The amount of PFAS sorbed onto beads at time *t* (*q*_*t*_) was determined using the following equation, where *C*_0_ is the initial concentration of PFAS solution (M), *C*_*t*_ is the concentration of PFAS solution at time *t* (M), *V* is the volume of the PFAS solution (L), and *m* is the dry mass of adsorbent (g),3
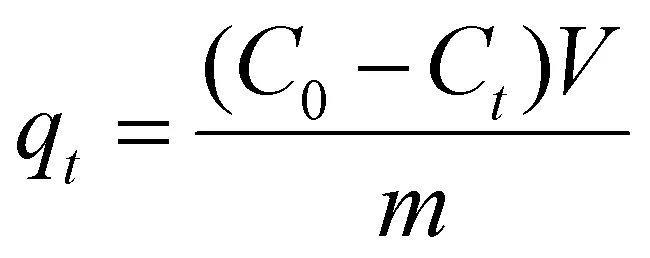


The intraparticle diffusion model (IPD, eqn (4)) was also used to assess the role of the PFAS diffusion in the sorption mechanism through the fitted intraparticle diffusion rate constant, *k*_d_ (mmol g^−1^ h^−0.5^):4
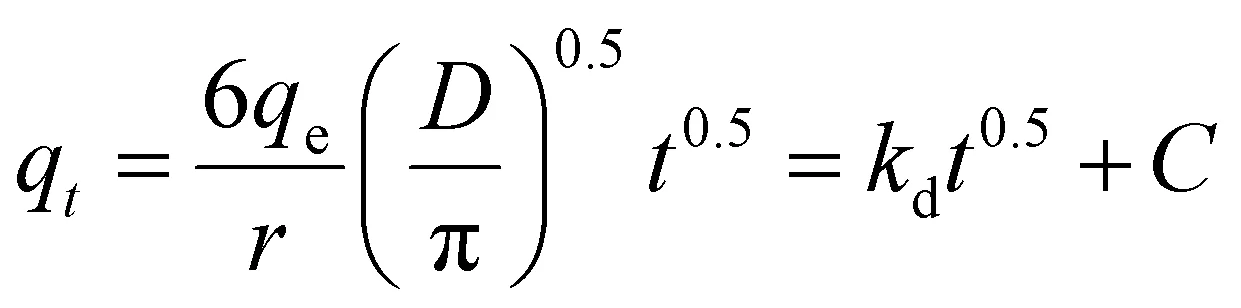
where *D* (mm^2^ h^−1^) is intraparticle diffusivity, *r* (mm) is the radius of the particle, *k*_d_ (mmol g^−1^ h^−0.5^) is the intraparticle diffusion rate constant, and *C* (mmol g^−1^) is the boundary layer thickness coefficient. More details of this model are provided in the SI.

#### 2.3.6. PFAS sorption isotherms

Equilibrium sorption of PFAS to PB-Q was compared to sorption to IRA 900 whole resin. For all experiments, 10 mg beads (without drying) per sample were used, and the isotherm data were corrected to the dry mass of the beads. A different vial was used for each concentration of the PFAS (TFA, PFBA, or PFOA) solutions (prepared in 100 mM phosphate buffer at pH 7) for the sorption isotherms. Each vial contained 10 mg beads and 16 mL PFAS solution. The vials were placed on an orbital shaker at 140 rpm for one week to ensure equilibrium. Experiments were conducted in duplicate.

Sorption isotherms were fit with the Langmuir and the Freundlich models (eqn (5) and (6)):5
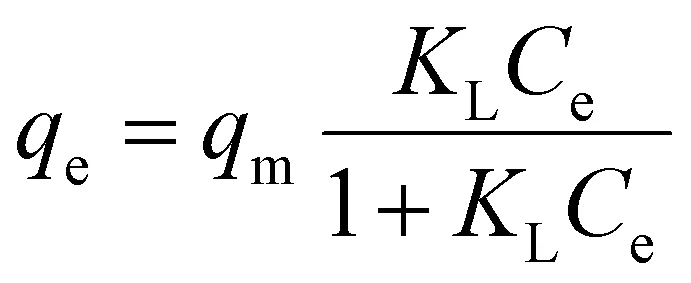

6 *q*_e_ = *K*_F_*C*^1/*n*^_e_

where *q*_e_ (mmol g^−1^) is the sorbed PFAS at equilibrium, *q*_m_ (mmol g^−1^) is the maximum sorption capacity of the adsorbent, *C*_e_ (mM) is the equilibrium concentration of PFAS in the solution, and *K*_L_ (L mmol^−1^) and *K*_F_ (mmol^(1−1/*n*)^ L^1/*n*^ g^−1^) are the equilibrium constants in the Langmuir and Freundlich isotherms, respectively. *K*_L_ quantitates affinity between adsorbate and adsorbent. A large *K*_L_ shows that adsorption is favorable even at low concentrations. In the Freundlich isotherm model, *n* represents sorption intensity. The Langmuir model assumes monolayer formation on a homogeneous surface, while the Freundlich model allows for multilayer formation on a heterogeneous surface.^39–41^

#### 2.3.7. PFAS desorption

First, 200 mg PB-Q were added to a 2 mM PFAS (TFA, PFBA, or PFOA) solution (prepared in 100 mM phosphate buffer at pH 7; 250 mL) to load the beads with PFAS. Then, the beads were removed from the PFAS solution and air-dried at room temperature. Four solvent systems (water, MeOH, 1 M NaCl_(aq)_, and a 1 : 1 (v/v) blend of MeOH and 1 M NaCl_(aq)_) were used to desorb the PFAS from the beads. In duplicate, 10 mg PFAS-loaded beads per 16 mL of a solvent system were added to glass vials. At room temperature, these vials were placed on an orbital shaker at 140 rpm for a week. The amount of sorbed PFAS on the beads was indirectly measured by determination of the remaining PFAS in the solution equilibrated with the beads using ^19^F-NMR spectroscopy and sorbed PFAS per unit mass of the beads (*q*_*t*_) was calculated using eqn (3). The amount of desorbed PFAS in the solvents was also measured by ^19^F-NMR spectroscopy and compared to *q*_*t*_.

#### 2.3.8. PFAS determination by ^19^F NMR spectroscopy

PFAS concentrations for the experiments described above were determined by ^19^F-NMR spectroscopy using a Bruker 600 MHz Avance Neo instrument equipped with a 5 mm triple resonance cryoprobe. Table S1 shows all the components of the NMR samples, and Table S2 gives the instrument settings. Settings were adjusted to allow complete relaxation of the ^19^F nuclei between pulses so that quantitative analysis could be performed.^42–44^ Fig. S6 shows the ^19^F-NMR spectra of PFAS (TFA, PFBA, and PFOA), 2,6-difluorophenol (DFP), and hexafluorobenzene (HFB). HFB was used as an internal reference for chemical shift, while DFP was used as an internal standard for quantification of PFAS. All these chemicals have distinct and non-overlapping NMR peaks. Integrals of NMR peaks of the –CF_3_ groups of PFAS were used for quantification.^45,46^ More detail on ^19^F-NMR spectroscopy analyses is given in the SI.

## 3. Results and discussion

### 3.1. Preparation of anion exchange beads

**Fig. 1:**
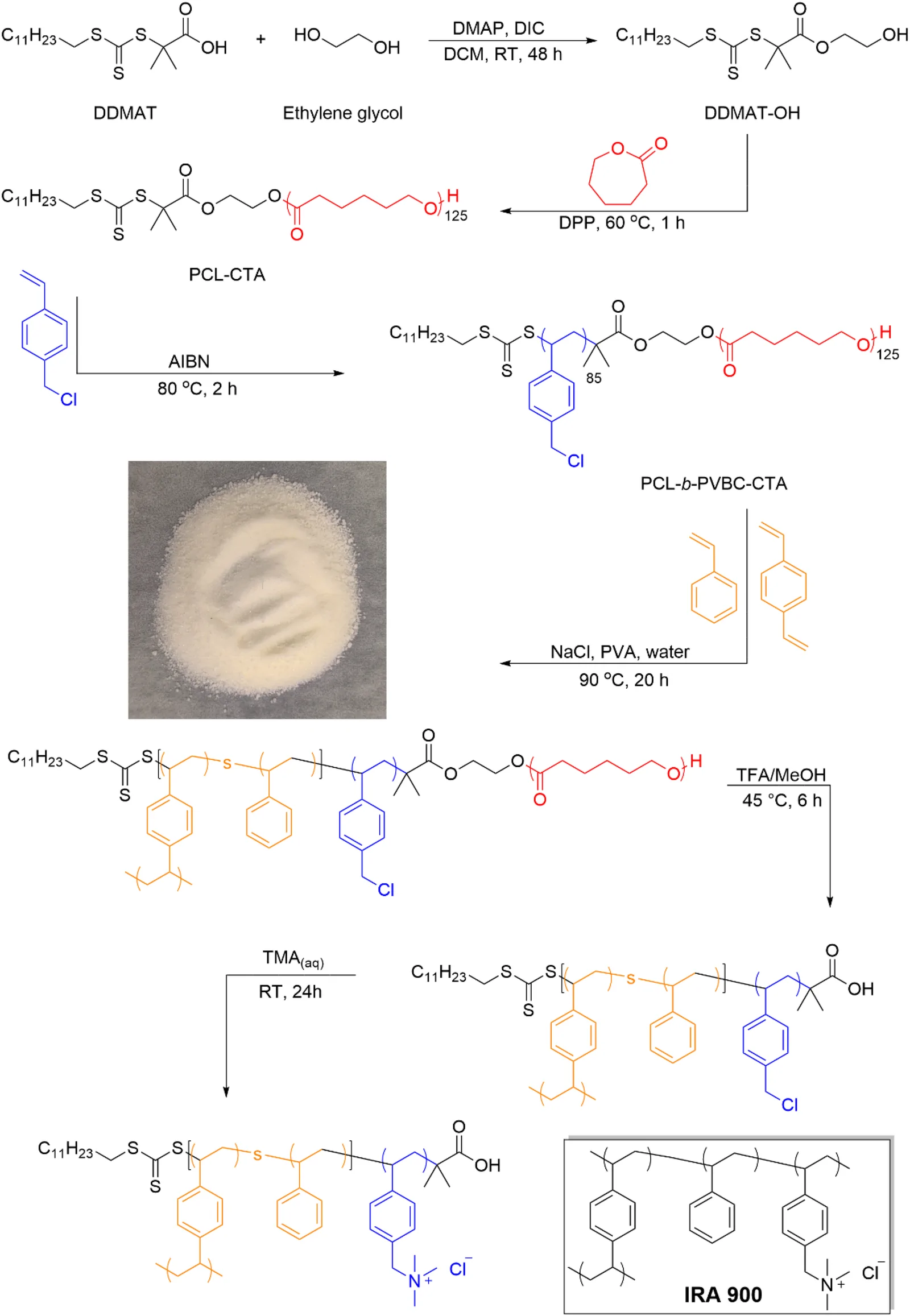
Reaction scheme of the synthesis of DDMAT-OH through esterification, PCL-CTA with ROP, PCL-*b*-PVBC-CTA by bulk RAFT polymerization, and beads through suspension RAFT polymerization, followed by acid etching of PCL and quaternization of PVBC. Box contains the chemical structure of IRA 900.

A PCL-*b*-PVBC diblock polymer was used to synthesize anion exchange beads. The PCL block was chosen as a sacrificial block to generate mesopores due to its ready degradability under acidic and basic conditions.^47^ The PVBC block was used as a precursor for generation of positive charges on the pore walls through post-polymerization quaternization at the benzyl carbon, as previously reported.^33,37^ First, 2-(dodecylthiocarbonothioylthio)-2-methylpropionic acid (DDMAT) was end-modified with ethylene glycol through esterification (Fig. 1). The resulting DDMAT-OH was then used as an initiator to prepare PCL homopolymer (PCL-CTA) through ROP using DPP as an acid catalyst.^48^ A PVBC block was then grown from the PCL-CTA through RAFT polymerization using AIBN as an initiator, giving PCL-*b*-PVBC-CTA.

Beads were synthesized using suspension RAFT polymerization (Fig. 1). We chose a 30 wt% loading of the PCL-*b*-PVBC-CTA and 70 wt% loading of styrene and divinylbenzene (2 : 1 mol ratio), targeting a 1 mmol g^−1^ charge density (assuming 100% conversion of all the benzyl chloride groups to quaternized ammonium groups) after all the post-polymerization modification steps. The size of the beads depends on the stirring rate during suspension polymerization,^33^ hence, the stirring rate was maintained at 600 rpm.

**Fig. 2:**
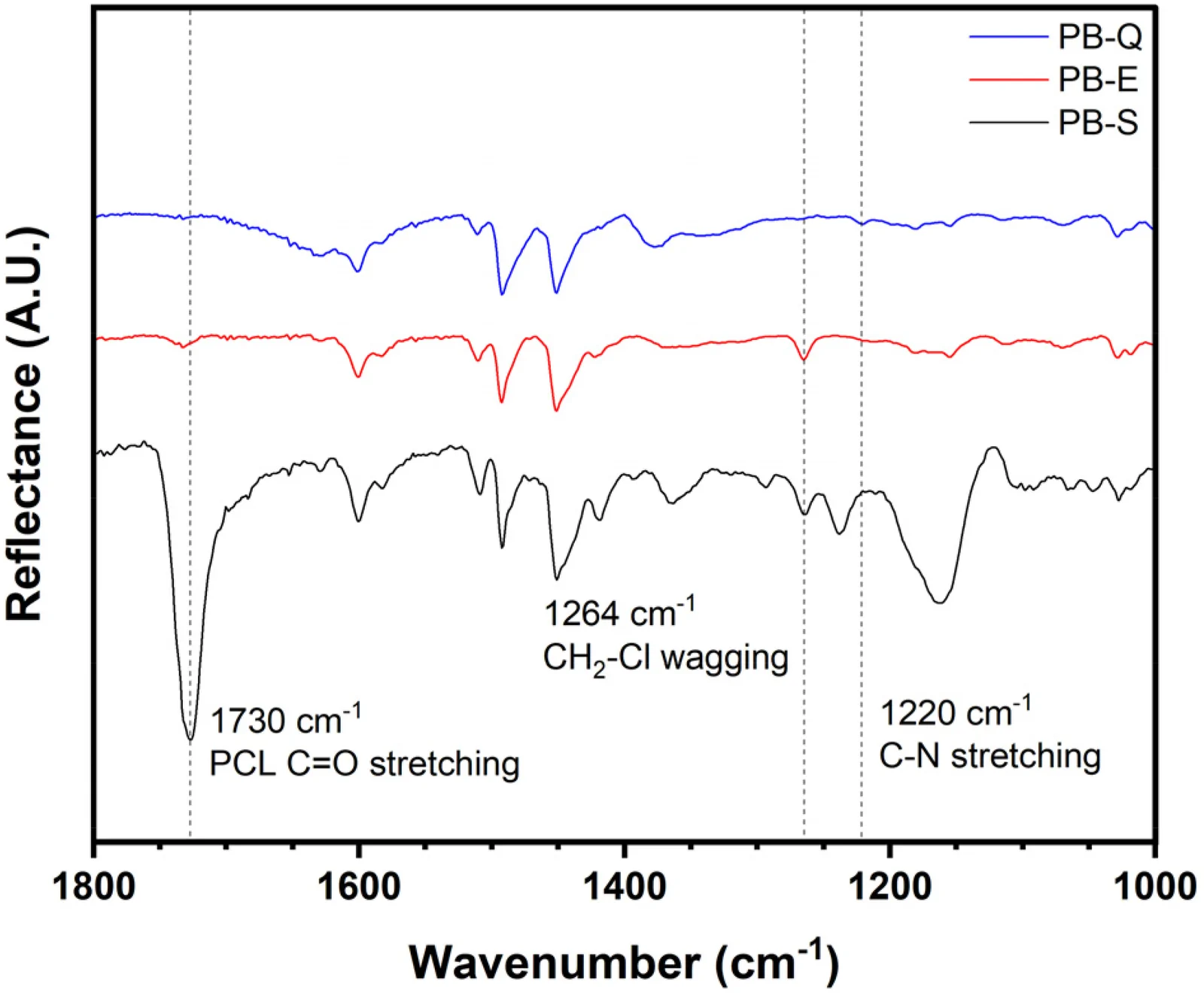
ATR-FTIR spectra of PB-S (black), PB-E (red), and PB-Q (blue). Peaks at 1730 cm^−1^ corresponding to C=O stretching in PCL, 1264 cm^−1^ for CH_2_–Cl wagging in PVBC, and 1220 cm^−1^ for C–N stretching in quaternized PVBC were observed.

The etching and quaternization of the beads were performed in two separate steps (Fig. 1). Alkaline conditions have been used for the etching of PCL; however, we observed a rather slow and incomplete etching of PCL in our specifically formulated beads, as confirmed by the presence of the C=O stretching peak of PCL at 1730 cm^−1^ (Fig. S7). Residual PCL increases the total weight of the bead, thereby reducing the ion exchange capacity (IEC) per unit mass. Moreover, C–Cl bonds in PVBC can undergo hydrolysis under alkaline conditions, as indicated by the decreased intensity of the CH_2_–Cl wagging peak at 1264 cm^−1^ (Fig. S7). The loss of C–Cl bonds reduces the number of available quaternization sites, also resulting in a lower IEC. Therefore, we optimized the PCL etching conditions and used a 1 : 1 (v/v) blend of trifluoroacetic acid (TFA) and MeOH.^35^Fig. 2 shows the ATR-FTIR spectra of as-synthesized (PB-S), etched (PB-E), and quaternized beads (PB-Q). Using TFA eliminates the side reactions, as shown by the absence of changes in the intensity of the PVBC CH_2_–Cl wagging signal in the FTIR spectrum of PB-E. Meanwhile, the C=O stretching peak of PCL was absent, suggesting complete etching of PCL.

In the second step, the treatment of PB-E with trimethylamine (TMA) resulted in the replacement of Cl with TMA, which gave the corresponding quaternary ammonium ion (–CH_2_–N^+^(CH_3_)_3_). We used TMA to obtain a small quaternary ammonium cation with a limited steric hinderance.^49^ In the ATR-FTIR spectrum of PB-Q (Fig. 2), the CH_2_–Cl wagging peak at 1264 cm^−1^ was absent, and a C–N stretching peak at 1220 cm^−1^ appeared, consistent with complete quaternization.

### 3.2. Bead analysis

**Fig. 3:**
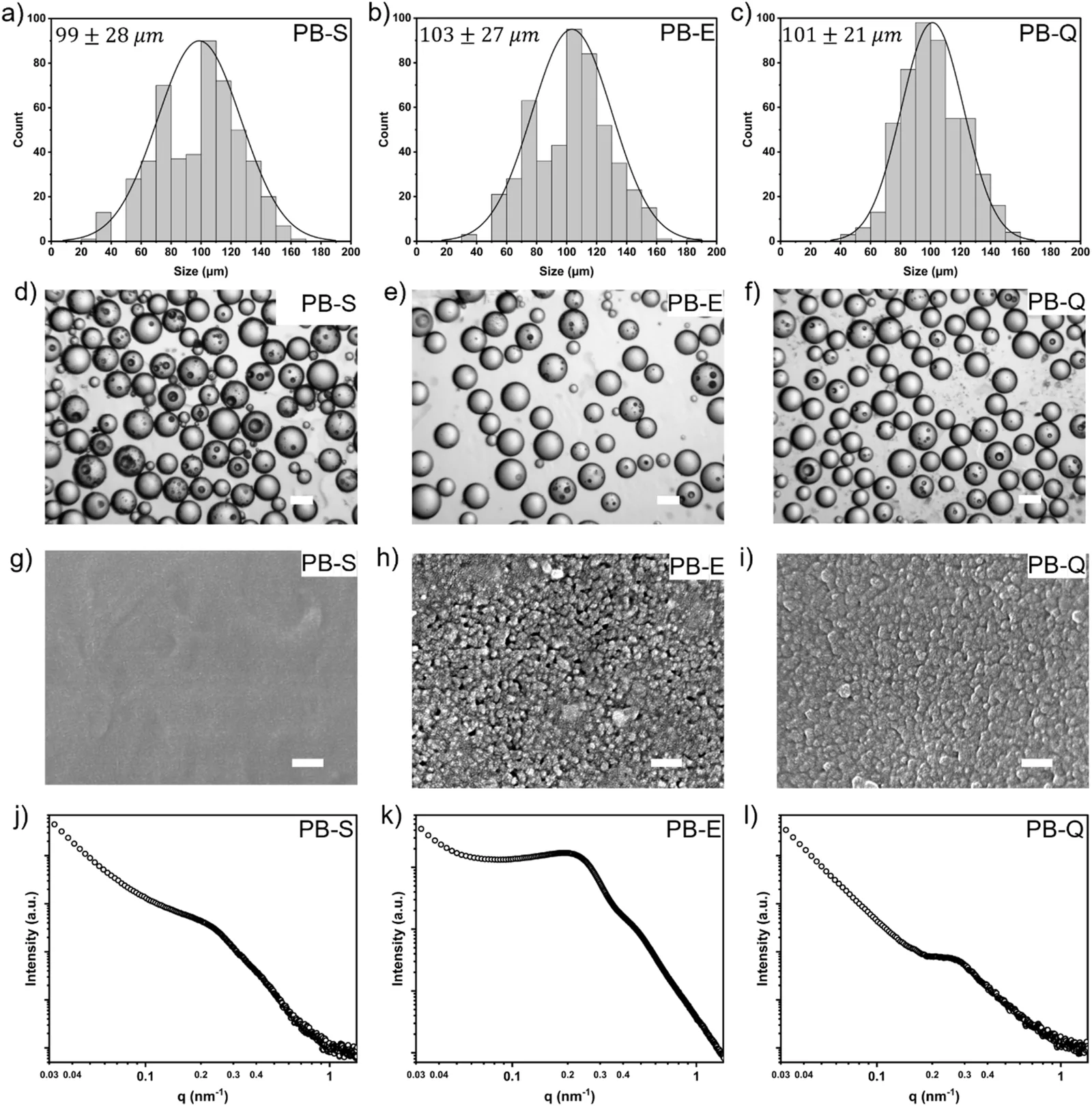
Bead size distribution (a–c) and optical microscope images with the highlighted scale of 100 µm (d–f) for PB-S with an average size of 99 ± 28 µm, PB-E with an average size of 103 ± 27 µm, and PB-Q with an average size of 101 ± 21 µm. SEM images (g–i) of the beads with highlighted scale of 100 nm. SAXS plots (j–l) of the beads with a broad scattering peak, suggesting microphase-separated disordered morphology.

Optical microscope images of the beads (Fig. 3d–f) showed spherical beads with diameters of about 100 µm. The average size of the beads did not change during the etching and quaternization steps (Fig. 3a–c).

Scanning electron microscope (SEM) images (Fig. 3g–i) of the beads were obtained to image the surface of the beads. As expected, PB-S had a smooth, nonporous surface, while PB-E had a porous surface. After quaternization, the apparent pore size decreased slightly, and the pore structure appeared somewhat constricted.

Small-angle X-ray scattering (SAXS) was used to confirm the microphase separation in the beads (Fig. 3j–l). PB-S was characterized by a broad scattering peak without higher-order features, suggesting a microphase-separated but disordered morphology.^50^ A scattering peak appeared at 0.23 nm^−1^, corresponding to a 27 nm domain spacing. The weak intensity of the scattering peak was likely due to a small electron density contrast between the phase-separated domains. The intensity of the scattering peak increased in PB-E due to the high electron contrast from the etching of the PCL domain, but the domain spacing remained unchanged. PB-Q also had the characteristic single broad scattering peak, consistent with a co-continuous morphology retained in the post-polymerization modifications. However, the scattering peak shifted to 0.26 nm^−1^ after quaternization, suggesting a decrease in domain spacing by 3 nm. The change in domain size may have arisen from swelling of the charged domains, resulting in a slight modification of the nanometer-scale morphology.

**Fig. 4:**
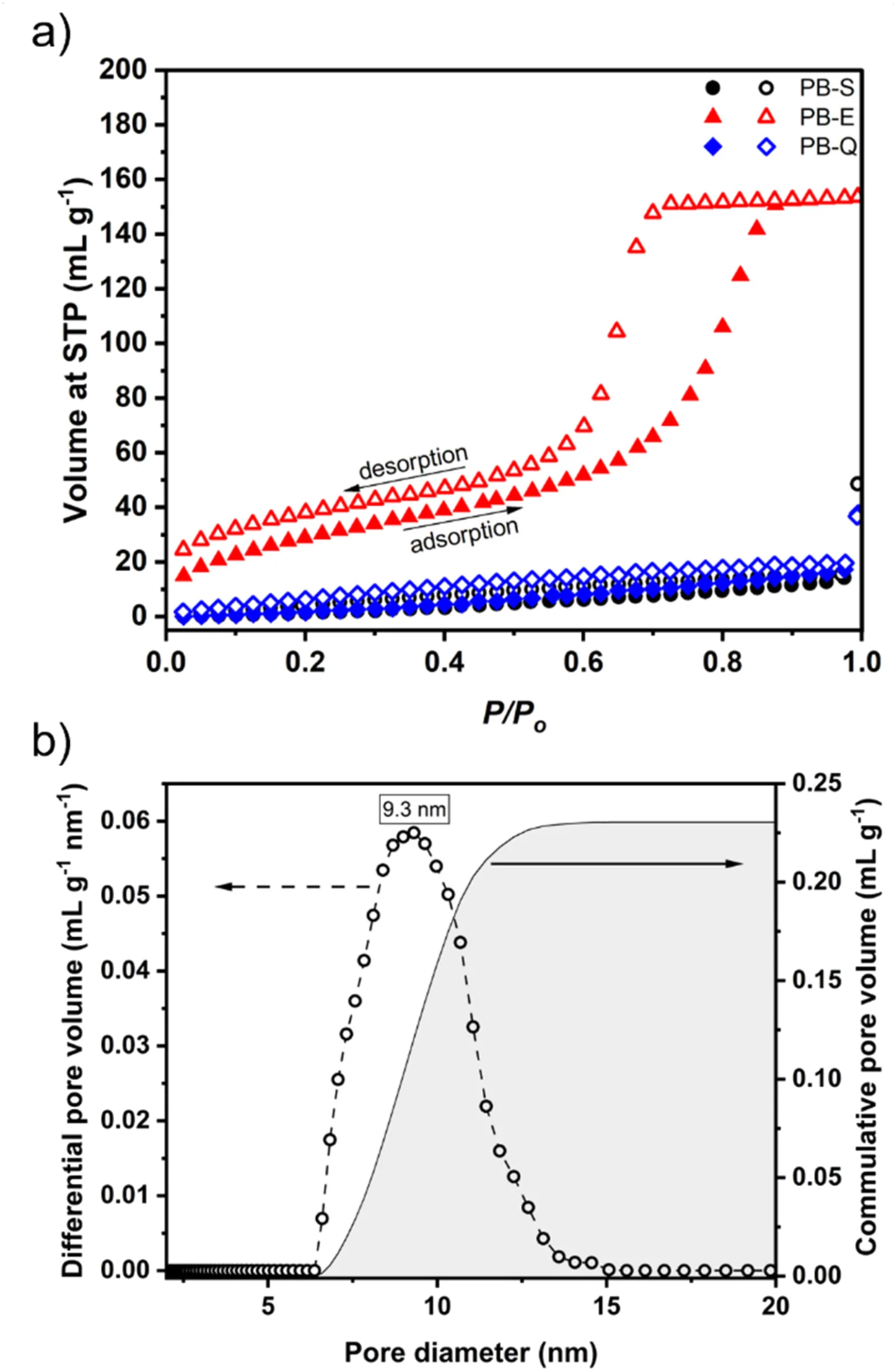
N_2_ sorption data of PB-S (black), PB-E (red), and PB-Q (blue). (a) Nitrogen sorption isotherms, with filled symbols for adsorption and empty symbols for desorption. (b) Pore size distribution in PB-E from the QSDFT model using the adsorption branch of the isotherm and assuming slit/cylindrical pores on the carbon surface. The mode diameter of the pores was 9.3 nm with 0.23 mL g^−1^ pore volume and 109 m^2^ g^−1^ surface, as obtained using the BET model.

Nitrogen sorption isotherms for all bead samples were collected to investigate the morphology and total surface area of the beads. Fig. 4a shows the sorption isotherms of PB-S, PB-E, and PB-Q. As expected, the sorption isotherm of PB-S showed no significant micro- or mesoporosity. The sorption isotherm of PB-E showed a type IV isotherm with low adsorption at low pressure, a high-pressure plateau, and associated hysteresis. This characteristic isotherm indicates pore-filling behavior in mesoporous materials.^51^ These data are consistent with the SEM image shown in Fig. 3h. After the quaternization of the beads, the isotherm of PB-Q was surprisingly very similar to that of PB-S, which is not consistent with mesoporosity retention post-quaternization by nitrogen adsorption, even though the SEM evidence in Fig. 3i suggests otherwise. Similar discrepancies between SEM data and nitrogen adsorption data have been observed in related materials. We posit that the polymer chains extend after quaternization and, in a hydrated state, fill the entire pore volume. As the effective length of the charged chains increases, the morphology of the materials appeared to transition from porous to non-porous.^52^ As the quaternized PVBC chains of the PB-Q beads seem to fill the mesopores, an adsorption isotherm results that resembles that of the PB-S beads. Brunauer–Emmett–Teller (BET) analysis was used to calculate the surface area of 109 m^2^ g^−1^ for PB-E. By using the quenched solid-state density functional theory (QSDFT) model on the adsorption branch of the isotherm and assuming slit/cylindrical pores on the carbon surface, the mode pore diameter was estimated to be approximately 9 nm, with 0.23 mL g^−1^ pore volume, confirming the mesoporosity (2–50 nm) in PB-E (Fig. 4b). However, the BET and QSDFT model analyses of PB-Q gave very low surface area and no porosity. The decrease in BET surface area and changes in mesopore distribution after quaternization are consistent with partial pore occupation or narrowing, caused by the newly introduced functional groups, as well as reduced accessibility of nitrogen to ion-containing pore environments.^52^ Meanwhile, Amberlite IRA 900, a macroporous resin, had a mode pore size of 50 nm by nitrogen adsorption analysis, with a surface area and pore volume of 25 m^2^ g^−1^ and 0.12 mL g^−1^, respectively (Fig. S8).

TGA showed that PB-S and PB-E were stable to significant mass loss until 300 °C (Fig. S9). However, PB-Q and IRA 900 contained approximately 5% and 57% water, respectively, which evaporated at ~100 °C. Then, there was an additional mass loss above 150 °C, likely corresponding to the decomposition of the quaternary ammonium groups by Hofmann elimination.

### 3.3. Ion exchange capacity (IEC)

Fig. S10 shows the conductometric titration data for PB-Q. Conductometric titration gave an IEC of 1.00 ± 0.05 mmol g^−1^ of dry beads, which is fully consistent with our targeted value. Fig. S11 shows the pH titration data upon equilibration of the hydroxide loaded beads with NaCl_(aq)_ solution. From the equivalence point of the titration curve, the number of OH^−^ ions and, thereby, the number of positive charges were calculated. This gave an IEC value of 1.01 mmol g^−1^ of dry PB-Q. The IEC of IRA 900 measured with the same pH titration method was 3.50 mmol g^−1^ of dry mass. More detail on the IEC measurement method is given in the SI.

### 3.4. Sorption kinetics

**Fig. 5:**
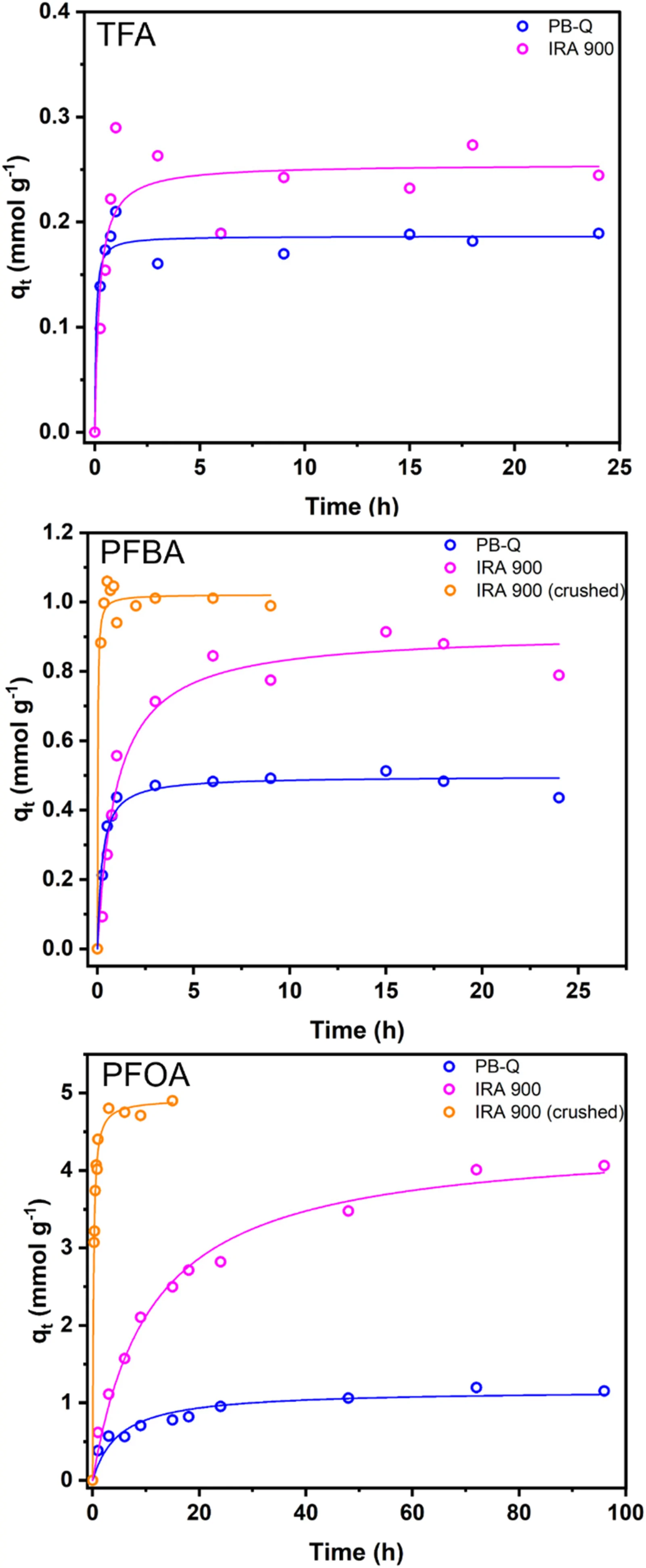
Sorption kinetics of PFAS (TFA, PFBA, or PFOA) with 10 mg (wet mass) PB-Q, IRA 900 and IRA 900 (crushed) separately in 16 mL of 2 mM PFAS solution for each data point. Values for *q*_*t*_ were corrected to the dry mass of the adsorbent. Averages of duplicates are shown. Solid lines represent the pseudo-second order kinetic model fitting.

We observed that PB-S and PB-E sorbed, as expected, little to no PFAS (Fig. S12), while sorption of PFAS to PB-Q and IRA 900 was a time dependent process (Fig. 5). The molar ratio of PFAS in the stock solution to the positive charges in the PB-Q and IRA 900 beads was greater than one. The PB-Q and IRA 900 data were fitted to evaluate the sorption kinetics using eqn (1) and (2). Saturation of PB-Q and whole IRA 900 occurred at 5 and 10 h for TFA and PFBA, respectively. For PFOA, the saturation point for PB-Q was 40 h, and whole IRA 900 did not reach saturation over 96 h. For both PFBA and PFOA, saturation was achieved much faster when the IRA 900 beads were crushed as compared to when IRA 900 beads were used directly (*e.g.*, for PFOA, saturation was reached at 6 h for crushed IRA 900 *versus* 4 d for whole IRA 900 beads). We posit that these faster kinetics resulted from the decreased particle size, which led to improved PFAS mass transfer to the more exposed binding sites. The kinetic data were fitted using pseudo-second order (Fig. 5) and pseudo-first order kinetic models (Fig. S13). Pseudo-second order and pseudo-first order fitting parameters are given in Table 1 and S3, respectively. With both models giving reasonably good fits, the pseudo-second order fitting was adopted for data analysis, because this model has been widely used for the sorption kinetics of PFAS to ion exchange materials.^39,40,53,54^

**Table 1:** Kinetic parameters and standard errors for TFA, PFBA, and PFOA sorption onto PB-Q and IRA 900, as obtained from fitting with the pseudo-second order model

Adsorbent	Adsorbate	Pseudo-second order parameters			
		*q*_e_ (mmol g^−1^)	*ν*_0_ (mmol g^−1^ h^−1^)	*k*_2_ (g mmol^−1^ h^−1^)	*R* ^2^
PB-Q	TFA	0.190 ± 0.007	3.61 ± 2.22	100 ± 61	0.92
	PFBA	0.500 ± 0.013	2.13 ± 0.42	8.5 ± 1.6	0.97
	PFOA	1.20 ± 0.07	0.24 ± 0.08	0.170 ± 0.054	0.92
IRA 900 (whole)	TFA	0.260 ± 0.018	1.22 ± 0.69	18 ± 10	0.80
	PFBA	0.910 ± 0.037	0.91 ± 0.21	1.10 ± 0.24	0.96
	PFOA	4.40 ± 0.14	0.41 ± 0.06	0.021 ± 0.003	0.99
IRA 900 (crushed)	PFBA	1.00 ± 0.02	58 ± 32	58 ± 32	0.98
	PFOA	4.90 ± 0.06	31.2 ± 2.5	1.3 ± 0.1	0.99

IRA 900 had higher predicted *q*_e_ values for all PFAS than those for PB-Q, as expected given that this resin has 3.5 times the number of positive charges per gram than PB-Q. Comparing *k*_2_ values of different adsorbents with varying IECs gives an incomplete understanding of their performance in removing adsorbates. *k*_2_ represents the overall sorption rate constant and can have a lower value for high ion exchange capacity materials if they take longer to reach equilibrium than adsorbents with lower ion exchange capacities and faster equilibrium times. The following equation (derived from eqn (2)) is another form of the pseudo-second order kinetic model, which provides *v*_0_ (mmol g^−1^ h^−1^) = *k*_2_*q*_e_^2^, an initial sorption rate:7
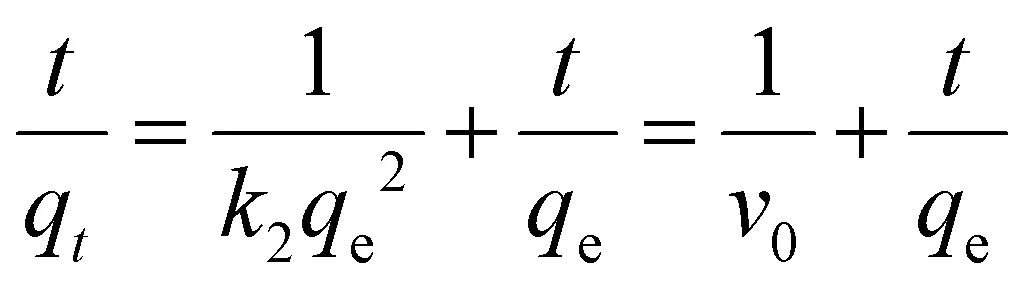


The initial sorption rate incorporates the pseudo-second order rate constant (*k*_2_) and equilibrium sorption capacity (*q*_e_) and better compares the efficiency of different adsorbents. It includes information about the uptake of adsorbates in the initial phase of the sorption process and is of more practical use where fast sorption is advantageous.

For PB-Q the initial sorption rates (*v*_0_) for TFA and PFBA were 2.9 and 2.3 times higher, respectively, than for whole IRA 900 beads (Table 1). In contrast, the initial sorption rate for PFOA was 1.7 times higher in case of whole bead IRA 900 than for PB-Q. The lower initial sorption rate of PFOA for PB-Q can be attributed to the larger size of the PFOA compared to TFA and PFBA, and, therefore, slower diffusion within the PB-Q beads. This may be the result of electrostatic repulsion-induced conformational changes of the linear PVBC chains within the pores of the PVBC, inhibiting faster diffusion. Notably, the initial sorption rate for PFBA and PFOA were 27 and 130 times larger, respectively, for crushed IRA 900 compared to PB-Q. Because the IRA 900 beads were crushed so that their particle size (Fig. S14) was similar to PB-Q bead size, we conclude that the difference in the observed sorption kinetics stems from difference in the morphology of the two materials. That is, the macroporous morphology of the IRA 900 and the gel-like morphology (resulting from putative pore restriction) of PB-Q. This is consistent with results from previous studies, which have shown that increasing the PFAS chain length reduces the initial sorption rate.^41^ This is primarily attributed to the larger molar volume of longer PFAS chains, which hinders both aqueous-phase and intra-particle diffusion. As a result, macroporous resins such as Amberlite IRA 910 and Amberlite IRA 958 exhibit faster initial sorption rates for compounds like PFOA and PFOS compared to gel-type resins such as Amberlite IRA 400 and Amberlite IRA 410.^41^

Due to pore size differences in adsorbents and the molecular size differences in the PFAS we explored, the diffusion of the PFAS in the beads likely plays an essential role in determining the characteristics of the sorption process. Table 2 contains all the parameters from fits of the experimental data with the IPD model, as shown in Fig. S14. *C* is the *y*-intercept and reports on the formation of a boundary layer that can influence the sorption mechanism. In all the fits, the data pass through the axes origin (*C* = 0) within error indicating that intraparticle diffusion is the only rate limiting step controlling the sorption mechanism. Comparing the *k*_d_ (intraparticle diffusion rate constant) values for PFAS sorption to PB-Q and IRA 900 (whole and crushed) provides insights into the relationship between pore size and PFAS chain length. During the initial phase of the sorption process, TFA sorption is characterized by similar *k*_d_ values for PB-Q and whole IRA 900. The *k*_d_ for PFBA was also similar for PB-Q and whole IRA 900. However, the intra-particle diffusion rate of PFOA in PB-Q was lower than that in whole IRA 900. This difference in the diffusion coefficient of PFOA can be attributed to the difference between the pore sizes of PB-Q and whole IRA 900, suggesting that the long-chain PFOA diffuses faster in the macropores of the whole IRA 900 than the restricted mesopores of PB-Q. The lower *k*_d_ values for PFOA sorption to PB-Q are consistent with the slower initial sorption rates for PB-Q as compared to whole IRA 900 (Table 1). Notably, an increase in the *k*_d_ values of PFBA and PFOA was observed in the case of crushed IRA 900 as compared to whole bead IRA 900. This can be explained by the dependence of *k*_d_ on the size of the adsorbent particles (eqn (4)). However, the intraparticle diffusion remained the rate limiting step even after crushing the IRA 900 resin.

**Table 2:** Intraparticle diffusion model parameters and standard errors for adsorbents (PB-Q and IRA 900) and PFAS (TFA, PFBA, and PFOA)

Adsorbent	Adsorbate	Intraparticle diffusion parameters		
		*k*_d_ a (mmol^−1^ g^−1^ h^−0.5^)	*C* (mmol g^−1^)	*R* ^2^
PB-Q	TFA	0.250 ± 0.030	0.003 ± 0.015	0.93
	PFBA	0.450 ± 0.033	0.002 ± 0.023	0.97
	PFOA	0.330 ± 0.041	0.016 ± 0.061	0.97
IRA 900 (whole)	TFA	0.260 ± 0.042	−0.008 ± 0.029	0.86
	PFBA	0.430 ± 0.031	−0.008 ± 0.031	0.95
	PFOA	0.660 ± 0.033	−0.019 ± 0.078	0.99
IRA 900 (crushed)	PFBA	2.2 ± 0.1	0.00 ± 0.04	0.99
	PFOA	5.6 ± 0.2	0 ± 0	0.98

a *k*_d_ values from the first linear fit of the IPD data are given.

### 3.5. Sorption isotherms

**Fig. 6:**
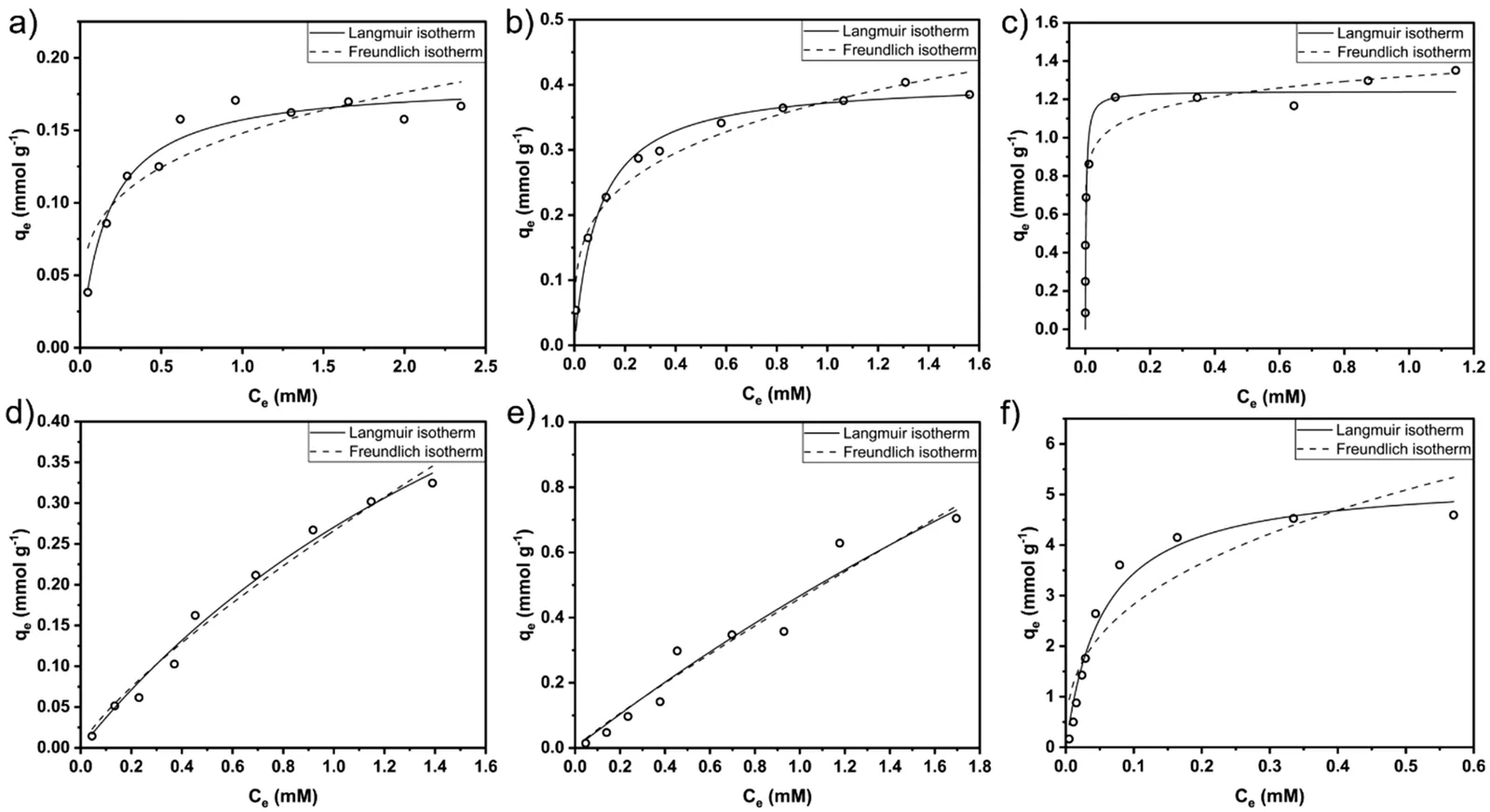
PFAS sorption isotherms of (a, d) TFA, (b, e) PFBA, and (c, f) PFOA for PB-Q (top row) and whole IRA 900 (bottom row). The solid lines represent Langmuir isotherm fits, and the dashed lines represent Freundlich fits.

Fig. 6(a–c) shows both Langmuir and Freundlich isotherms for TFA, PFBA, and PFOA sorption to PB-Q. The Langmuir isotherms (shown solid) provide the sorption capacities of PB-Q for TFA, PFBA, and PFOA as 0.180, 0.410, and 1.20 mmol g^−1^, respectively (Table 3). These values are similar to the *q*_e_ values shown in Table 1 from the kinetic experiments, indicating that full capacity of the beads was reached within the timeframe of the kinetic experiments, as PB-Q did not uptake more PFAS over the longer time scale of the isotherm experiments. Because the IEC of the beads is 1.00 mmol g^−1^, 1.00 mmol g^−1^ of PFAS sorbing onto the beads would be expected assuming only an ion exchange process. However, the PFOA sorption capacity was higher than the IEC, which suggests that both ion exchange and van der Waals interactions are involved in the sorption of long-chain PFAS to PB-Q. On the other hand, the *q*_m_ values for both TFA and PFBA were lower than the IEC of PB-Q. This discrepancy might be due to factors such as anion exchange competition or inaccessibility of PFAS to functional sites.

**Table 3:** Langmuir isotherm parameters and standard errors for TFA, PFBA, and PFOA sorption on PB-Q and IRA 900

Adsorbent	Adsorbate	Langmuir parameters		
		*q*_m_ (mmol g^−1^)	*K*_L_ (L mmol^−1^)	*R* ^2^
PB-Q	TFA	0.180 ± 0.006	5.9 ± 1.0	0.95
	PFBA	0.410 ± 0.011	11.0 ± 1.5	0.98
	PFOA	1.20 ± 0.09	390 ± 230	0.82
IRA 900 (whole)	TFA	0.91 ± 0.20	0.42 ± 0.13	0.98
	PFBA	3.8 ± 3.7	0.14 ± 0.16	0.94
	PFOA	5.30 ± 0.29	18 ± 3	0.97

Fig. 6(d–f) shows the sorption isotherms using whole IRA 900. Whole IRA 900 sorption capacities for PFAS were higher than those obtained from the pseudo-second order kinetic model (Table 1). This discrepancy in the sorption capacities is explained by the weak affinity of PFAS for whole IRA 900 (given by *K*_L_ in the Langmuir model and *n* in the Freundlich model; Tables 3 and S4). In short-time-scale kinetic experiments, the sorption capacities were low; however, in long-time-scale isotherm experiments, more PFAS molecules could sorb on the whole IRA 900 despite its lower affinities for PFAS. Isotherms were not generated for the crushed IRA 900, but we assume that at equilibrium the isotherms for whole bead and crushed IRA 900 would be within error identical.

A comparison between the Langmuir sorption isotherm of PB-Q and IRA 900 indicates that the affinity of PB-Q for both short-chain (TFA and PFBA) and long-chain PFAS (PFOA) is stronger than that for IRA 900. Despite lower initial sorption rates of PFOA in PB-Q than in IRA 900, at equilibrium PB-Q provides more efficient removal of TFA, PFBA, and PFOA. For example, from the *K*_L_ of 390 L mmol^−1^ and the Langmuir isotherm equation it follows that with a large excess of sample, half saturation of PB-Q beads is reached at a PFOA sample concentration of 2.6 × 10^−6^ M (1.1 ppm). In contrast, it takes 56 × 10^−6^ M (23 ppm) PFOA to reach half saturation of whole IRA 900. A similar ratio of the half saturation points is obtained for TFA (19 ppm and 238 ppm for PB-Q and whole IRA 900, respectively.) Interestingly, the increased affinity of PB-Q *versus* whole IRA 900 is most pronounced for PFBA, with the half saturation points at 19 ppm and 1.5 × 10^3^ ppm, respectively.

The difference in the affinities may arise from multiple factors. First, the PFAS removal efficiency is strongly related to the pore size of adsorbents, and the optimal pore size was found to be 2.5–4 times the PFAS molecular size.^24^ Thus, the large pore size (50 nm) of IRA 900 can negatively affect its affinity for the PFAS molecules by reducing the effective contact between PFAS molecules and the sorption sites and weakening the electrostatic interactions. Second, PB-Q had positively charged linear chains that effectively interact with PFAS molecules, unlike in IRA 900, where charged groups are present on the pore walls. PB-Q and IRA 900 contain the same functional group but differ in their PFAS uptake capacity. IRA 900 has 3.5 times more positive charges per gram. However, the ratio of sorbed PFOA molecules to available charges is similar for both IRA 900 and PB-Q.

**Fig. 7:**
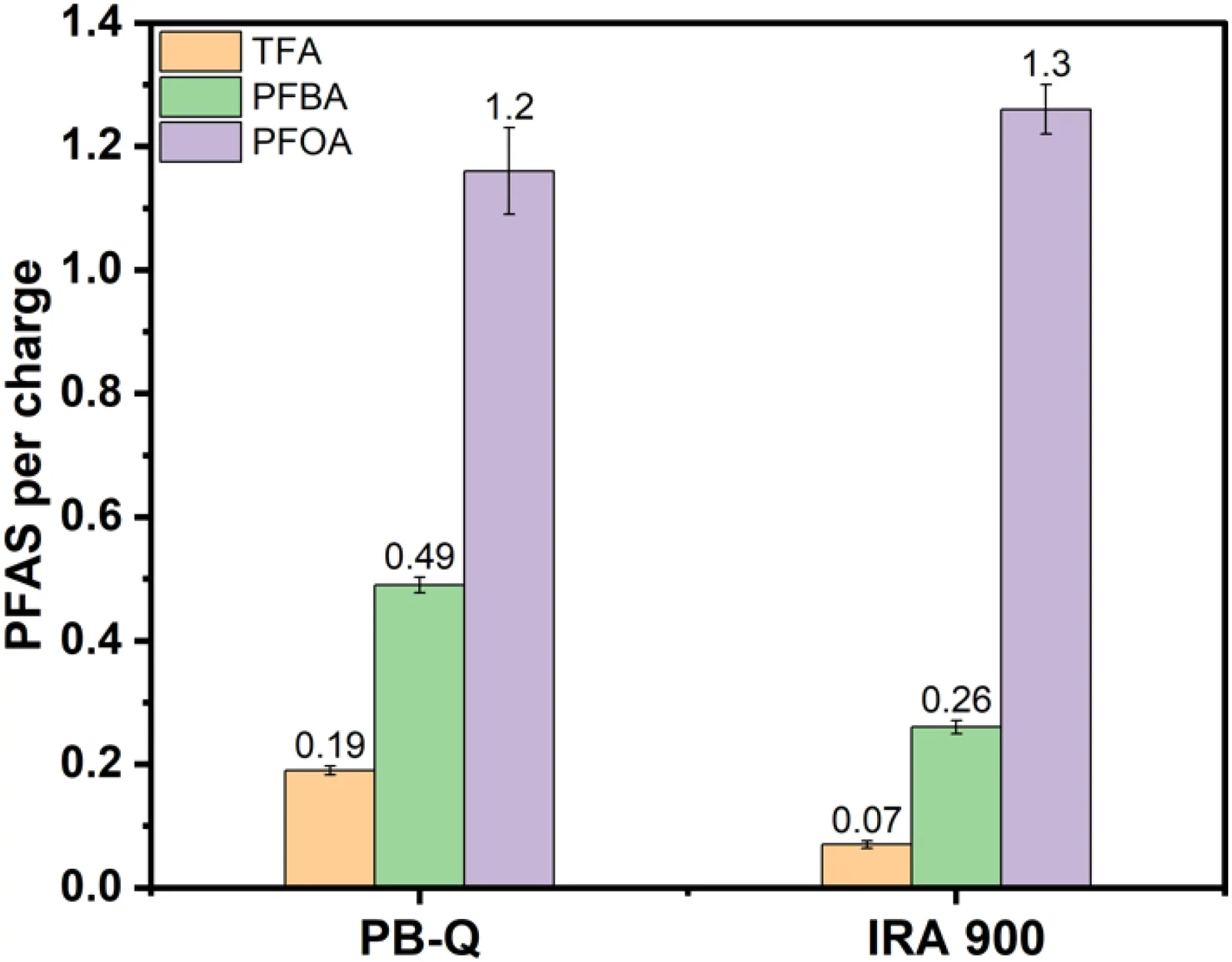
PFAS sorbed per charge (–CH_2_–N^+^(CH_3_)_3_ group) on PB-Q and IRA 900. PB-Q showed better PFAS to charge sorption for TFA and PFBA than whole IRA 900, while PFOA to charge sorption was similar for both adsorbents.

Fig. 7 compares PFAS sorption per charge (–CH_2_–N^+^(CH_3_)_3_ group) for PB-Q and whole IRA 900. Because the IEC of PB-Q is 1.00 mmol g^−1^ and IRA 900 is 3.50 mmol g^−1^, sorbed PFAS per charge provides additional mechanistic insight into the performance of PB-Q and whole IRA 900. For TFA and PFBA, PB-Q took up 0.19 and 0.49 molecules per charge, respectively. This is compared to 0.07 and 0.26 molecules per charge using whole IRA 900, for TFA and PFBA, respectively. PFOA sorption per charge was in the same range for PB-Q and whole IRA 900. While the type of functional groups is the same in PB-Q and whole IRA 900, the positive charges of IRA 900 are located on the walls of its macropores whereas in PB-Q those charges are situated on the dangling polymer chains that fill the mesopores.

### 3.6. PFAS desorption

**Fig. 8:**
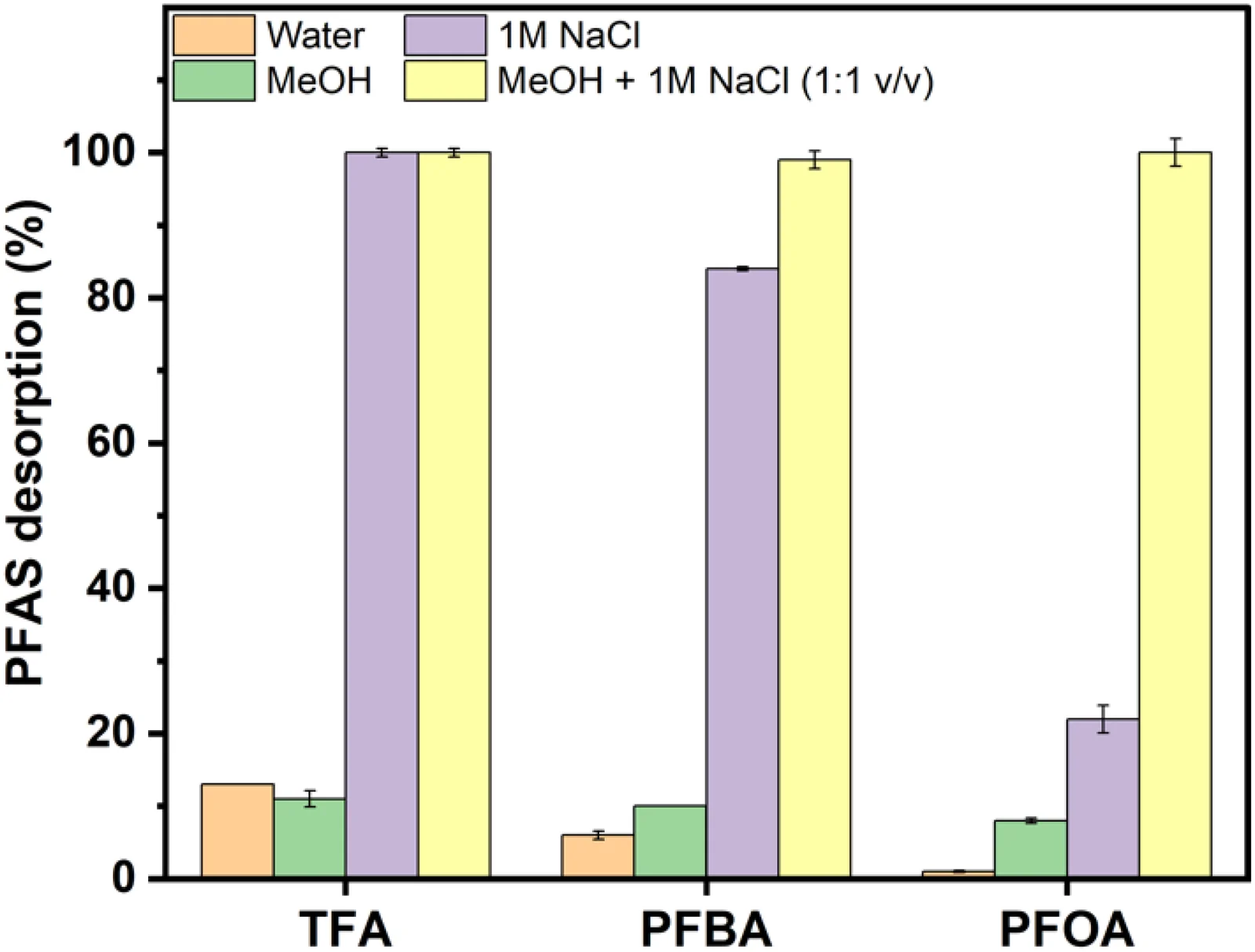
Desorption of PFAS from PB-Q using water, methanol (MeOH), 1 M NaCl, and MeOH in 1 M NaCl (1 : 1 v/v).

Many different solution systems with varying concentrations have been reported in the literature to desorb PFAS from used ion exchange resins, including NaCl_(aq)_, KCl_(aq)_, NH_4_Cl_(aq)_, NaOH_(aq)_, methanol (MeOH), and ethanol.^55,56^Fig. 8 illustrates PFAS desorption from PB-Q using water, methanol, 1 M NaCl_(aq)_, and a 1 : 1 v/v mixture of methanol and 1 M NaCl_(aq)_. For each of these solvents, 10 mg PFAS loaded beads per 16 mL of the solvent were used. PFAS desorption with water was 13%, 6%, and 1% for TFA, PFBA, and PFOA, respectively. MeOH showed a similar desorption trend with 11%, 10%, and 8% desorption of TFA, PFBA, and PFOA, respectively. 1 M NaCl_(aq)_ solution demonstrated 100% desorption for TFA, 84% for PFBA, and 22% for PFOA. Complete desorption for all three PFAS was achieved using a (1 : 1 v/v) solution of methanol and aqueous 1 M NaCl_(aq)_. This combination likely worked synergistically, with methanol weakening the van der Waals interactions between PFAS and the beads, and NaCl providing a high concentration of Cl^−^ to displace PFAS through competitive anion exchange. This PFAS desorption data agree with reported solution systems (a combination of salt and organic solvent) that show efficient desorption.^41^ This suggests that PFAS captured by PB-Q can be recovered for further processing/destruction.

## 4. Conclusions

In this study, we developed a new synthetic route to make PFAS-capturing anion exchange materials using polymerization-induced microphase separation. As-synthesized beads were etched in acidic conditions and subsequently quaternized. The shape and morphology of the obtained beads did not change during post-synthesis modifications. Quaternized beads exhibited improved PFAS sorption as compared to whole IRA 900 when tested against three different PFAS compounds. Particularly, they demonstrated higher PFAS uptake per positive charge, stronger adsorbate–adsorbent affinity, and faster initial sorption rates except for PFOA uptake, which was slower than that for whole IRA 900. While the PB-Q resin tested here was not rigorously optimized for ion exchange capacity, a key advantage of the PIMS synthetic approach is the ability to tune IEC and other key material parameters including pore size. Future work may improve ion exchange capacity for PB-Q by carefully iterating on the design and molecular weight of the quaternized polymer. Since such chemical and morphological factors strongly affect PFAS diffusion into the porous matrix and thus initial sorption rates, pore optimization may improve the uptake performance of quaternized beads for long-chain PFAS, building on the strong affinities of PFAS for PB-Q demonstrated here. Optimizing the chemical structure of the quaternized beads using PIMS, along with assessing their PFAS removal performance across a range of PFAS and evaluating their reusability, represents a critical step toward translating this bead synthesis strategy to scalable water treatment applications.

## Author contributions

Ali Arshad: conceptualization, investigation, manuscript writing (original draft), reviewing and editing. Jongho Back: data collection and analysis, manuscript writing, reviewing and editing. Katharine A. Faber: ^19^F-NMR spectroscopy method development, manuscript reviewing and editing. William A. Arnold: funding acquisition, advising, manuscript writing, reviewing and editing. Philippe Bühlmann: conceptualization, advising, access to laboratory facilities, manuscript writing, reviewing and editing. Marc A. Hillmyer: conceptualization, advising, access to laboratory facilities, manuscript writing, reviewing and editing.

## Conflicts of interest

The authors declare no competing financial interests.

## Supplementary Material

LP-004-D6LP00075D-s001

## Data Availability

The raw data files used in this manuscript are openly available in the Data Repository for University of Minnesota (DRUM) at https://hdl.handle.net/11299/166578. Supplementary information (SI) is available. See DOI: https://doi.org/10.1039/d6lp00075d.
