# Supplementary material for: Anion exchange beads for PFAS capture using a polymerization-induced microphase separation approach
Source: RSC Appl Polym. 2026 Jun 17;4(5):1596–610. doi: 10.1039/d6lp00075d (PMC13274420; doi:10.1039/d6lp00075d)
Supplement: Supplementary file 2 [file LP-004-D6LP00075D-s001.pdf]

Supplementary Information (SI) for RSC Applied Polymers.
This journal is © The Royal Society of Chemistry 2026
Supplementary Information (SI) for RSC Applied Polymers.
This journal is © The Royal Society of Chemistry

Supporting information for

Anion Exchange Beads For PFAS Capture Using a Polymerization-Induced Microphase Separation Approach

Ali Arshad,[a] Jongho Back,[a] Katharine A. Faber,[b] William A. Arnold,[b,*] Philippe Bühlmann,[a,*] and Marc A. Hillmyer[a,*]

[a] *Department of Chemistry, 207 Pleasant St. SE, University of Minnesota, Minneapolis, MN 55455-0431, USA*

[b] *Department of Civil, Environmental and Geo-Engineering, 500 Pillsbury Dr. SE, University of Minnesota, Minneapolis, MN 55455- 0431, USA*

*Corresponding authors (e-mail: arnol032@umn.edu, buhlmann@umn.edu, hillmyer@umn.edu)

Table of Contents

Synthesis of the DDMAT-OH S6

Synthesis of the PCL-CTA S6

Synthesis of PCL-b-PVBC-CTA copolymer..S7

Fluorine-19 NMR conditions and parameters S8

Intraparticle diffusion model (IPD)...S10

2. Supplemental Results S11

Polymers syntheses S11

Bead characterization S17

Ion exchange capacity (IEC) measurements S20

Kinetic data and fitting S23

References S27

Figures

Figure S1. The ^{1}H-NMR spectrum of DDMAT in $CDCl_3$ with all the peaks labelled and integrated. S11

Figure S2. The ^{1}H-NMR spectrum of DDMAT-OH in $CDCl_3$ with all the peaks labelled and integrated. S12

Figure S3. The ^{1}H-NMR spectrum of PCL-CTA in $CDCl_3$ with all the peaks labelled and integrated. S13

Figure S4. The ^{1}H-NMR spectrum of PCL-b-PVBC-CTA in $CDCl_3$ with all the peaks labelled and integrated. S14
Figure S5. THF-SEC dRI (normalized) traces of PCL-CTA and PCL-b-PVBC-CTA. After adding a PVBC block to the PCL homopolymer, the SEC trace shifted to a lower elution time (high molecular weight). S15
Figure S6. ^{19}F-NMR spectra of TFA, PFBA, and PFOA with DFP and HFB as internal standards. Integrals of the characteristic $–CF_3$ peaks (–76 ppm to –83 ppm versus $–CCl_3F$) were used for quantitative analysis. S16
Figure S7. ATR-FTIR spectra of PB-S (black) and alkaline-etched beads after 6 days (red). The 1730 cm^{-1} peak corresponds to C=O stretching in PCL: the 1264 cm^{-1} peak results from CH_2-Cl wagging in PVBC. S17
Figure S8. N_2 sorption data for IRA 900. (a) Nitrogen sorption isotherms with filled symbols for adsorption and empty symbols for desorption. (b) Pore size distribution using the QSDFT model using the adsorption branch of the isotherm and assuming slit/cylindrical pores on the carbon surface. The mode diameter of the pores was 50 nm, with 0.12 mL g^{-1} pore volume and 25 m^2 g^{-1} surface, as obtained with the BET model. S18
Figure S9. TGA traces of PB-S (black) and PB-E (red), PB-Q (blue), and IRA 900 (magenta). PB-S and PB-E were dry and stable below 300 °C. PB-Q and IRA 900 comprised 5% and 57% water, respectively, which evaporated at around 100 °C. The mass loss after 150 °C is attributed to the loss of quaternary ammonium groups in the quaternized beads. S19
Figure S10. Conductometric titration graph for ion exchange capacity (IEC) measurement of PB-Q. The conductivity of the bead suspension in the deionized water did not increase until all the $Cl^–$ ions had reacted with Ag^+. S21

Figure S11. pH titration graph for IEC measurement of PB-Q. The exchange of Cl^- counter ions with OH^- ions (using $NaOH_{(aq)}$) followed by another exchange of OH^- with Cl^- (using NaCl) gave a basic $NaCl_{(aq)}$ solution. The OH^- ions in the $NaCl_{(aq)}$ solution were titrated against 0.01 N $HCl_{(aq)}$. S22

Figure S12. Sorption of PFAS vs time using PB-S (black), PB-E (red), and PB-Q (blue), as well as IRA 900 (magenta). a) TFA, b) PFBA, and c) PFOA. For all data points, 10 mg wet beads were added to 16 mL of 2 mM PFAS (TFA, PFBA, or PFOA) solution in a glass vial, and vials were placed on an orbital shaker at 140 rpm at room temperature. S23

Figure S13. Sorption kinetics of PFAS (TFA, PFBA, or PFOA) with 10 mg (wet mass) PB-Q, IRA900 and IRA 900 (crushed) separately in 16 mL of 2 mM PFAS solution for each data point. Values for q_t were corrected to the dry mass of the adsorbent. Averages of the duplicates were plotted. Solid lines represent pseudo-firs order (PFO) kinetic model fitting. S23

Figure S14. Digital microscopic image of IRA 900 (crushed) with scale bar representing 100 µm. S25

Figure S15. IPD model fitting PFAS sorption kinetics data from a batch sorption experiment. S25

Tables

Table S1. Composition of the samples prepared for ^{19}F-NMR spectroscopy. The solutions were prepared in 2 mL glass vials before transferring them to NMR tubes. S9

Table S2. NMR spectroscopy parameters for all ^{19}F-NMR spectra. Bruker 600 MHz NMR instrument was configured to these settings for all quantitative ^{19}F-NMR spectral analyses. S9

Table S3. Kinetic parameters of the PFO model for the sorption of TFA, PFBA, and PFOA to PB-Q, IRA 900 and IRA 900 (crushed)....................S24

Table S4. Freundlich isotherm parameters for sorption of TFA, PFBA, and PFOA on PB-Q and IRA 900. ....S26

1. Supplemental Methods

Synthesis of the DDMAT-OH

2-(Dodecylthiocarbonothioylthio)-2-methylpropionic acid (DDMAT) was synthesized using a previously reported method.[1] As in the previously reported esterification procedure, DDMAT was end-modified using ethylene glycol.[2] DDMAT (16.5 g, 0.045 mol, 1 eq.) and 4-dimethylamino pyridine (DMAP; 0.5 g, 0.004 mol, 0.09 eq.) were dissolved in 200 mL dichloromethane (DCM) in a flame-dried 1 L round-bottom flask. Under argon, diisopropylcarbodiimide (DIC; 50 mL, 0.32 mol, 7 eq.) was added to the flask, turning the solution turbid. The solution was stirred at room temperature for 20 min, and ethylene glycol (255 mL, 4.6 mol, 100 eq.) was added. The reaction solution was stirred at room temperature for 48 h. After the reaction, the solution was diluted by adding 100 mL DCM, followed by washing with water (200 mL), an aqueous solution of copper(II) sulfate (0.06 M, 200 mL), and a saturated solution of sodium bicarbonate (2 × 200 mL), and brine (200 mL). The organic phase was dried over magnesium sulfate ($MgSO_4$), and the solvent was evaporated under reduced pressure. The crude product was purified using column chromatography (hexane/ethyl acetate 2:1), followed by solvent removal using a rotary evaporator. The purified product was a yellow-orange oil with a 65% yield. ^{1}H NMR (400 MHz, $CDCl_3$, **Figure S2**): δ 4.29 – 4.21 (m, 2H), 3.84 – 3.76 (m, 2H), 3.31 – 3.23 (m, 2H), 1.71 (s, 6H), 1.68 – 1.60 (m, 2H), 1.43 – 1.33 (m, 2H), 1.33 – 1.21 (m, 16H), 0.87 (t, *J* = 6.8 Hz, 3H).

Synthesis of the PCL-CTA

PCL macro-CTA (PCL-CTA) was synthesized by ring-opening polymerization of ε-caprolactone. DDMAT-OH (4.12 g, 0.010 mol, 1 eq.), distilled ε-caprolactone (115.1 g, 1.0 mol, 100 eq.), and

diphenyl phosphate (DPP; 5.04 g, 0.020 mol, 2 eq.) were added to a flame-dried 250 mL round-bottom flask. The reaction mixture was stirred in an oil bath at 60 °C until the stirring stopped due to the high viscosity of the mixture (approximately 1 h). The reaction mixture was then diluted with 100 mL of chloroform, and the reaction was quenched by adding 1 mL of pyridine at room temperature. The polymer was precipitated in methanol and redissolved in tetrahydrofuran (THF), followed by a second precipitation in hexane. The solid polymer (yellow powder) was recovered and dried in a vacuum oven at 40 °C overnight ($M_{\mathrm{n,\,NMR}}$ = 14 kg mol^{-1}, $M_{\mathrm{n,\,SEC}}$ = 9 kg mol^{-1}, $M_{\mathrm{w,\,SEC}}$ = 11 kg mol^{-1}, $Đ$ = 1.24). ^{1}H NMR (400 MHz, $CDCl_3$, **Figure S3**) δ 4.27 (dtd, J = 7.5, 5.2, 2.8 Hz, 4H), 4.03 (t, J = 6.7 Hz, 248H), 3.62 (t, J = 6.5 Hz, 2H), 3.24 (t, J = 7.4 Hz, 2H), 2.28 (t, J = 7.5 Hz, 252H), 1.62 (dtd, J = 14.9, 7.2, 3.8 Hz, 511H), 1.49 – 1.29 (m, 257H), 1.23 (s, 21H), 0.85 (t, J = 6.7 Hz, 4H).

Synthesis of PCL-b-PVBC-CTA copolymer

For the synthesis of diblock macro-CTA, PCL-CTA (7 g, 0.00050 mol, 1 eq.) was dissolved in 4-vinylbenzyl chloride (4-VBC, 23 g, 0.15 mol, 296 eq.) in a flame-dried 100 mL round-bottom flask. AIBN (0.025 g, 0.00015 mol, 0.30 eq.) was added as an initiator for RAFT polymerization of 4-VBC onto the PCL chain. The solution was degassed by bubbling dry nitrogen through it for 15 min. The reaction solution was stirred at 80 °C for 2 h. After the polymerization, the solution was diluted with 50 mL THF, and the product was precipitated twice in methanol. The solid product was dried under a vacuum at 40 °C overnight. The polymer (PCL-*b*-PVBC-CTA) was a yellow powder ($M_{\mathrm{n,\,NMR}}$ = 27 kg mol^{-1}, $M_{\mathrm{n,\,SEC}}$ = 18 kg mol^{-1}, $M_{\mathrm{w,\,SEC}}$ = 21 kg mol^{-1}, $Đ$ = 1.15; **Figures S4-S5**). ^{1}H NMR (400 MHz, $CDCl_3$) δ 7.07 (d, J = 27.1 Hz, 177H), 6.41 (q, J = 38.5 Hz, 171H), 4.67 – 4.31 (m, 169H), 4.05 (t, J = 6.7 Hz, 248H), 3.63 (t, J = 6.5 Hz, 4H), 3.31 – 3.14 (m, 2H), 2.29 (t, J = 7.5 Hz, 259H), 1.64 (dtt, J = 14.9, 10.3, 6.2 Hz, 581H), 1.53 – 1.27 (m, 393H).

Fluorine-19 NMR conditions and parameters

^{19}F-NMR analysis was used for the quantification of PFAS in all aqueous samples using a modified version of previously published experiment.[3] $Cr(acac)_3$ was used as relaxation agent instead of $Fe(acac)_3$ because of the poor solubility of $Fe(acac)_3$ in phosphate buffer. The final concentration of the relaxation agent was kept at 5 mM. Sample preparations were done in 2 mL glass vials. D_2O, hexafluorobenzene (HFB), 2,6-difluorophenol (DFP), and $Cr(acac)_3$ solutions were added to the glass vial followed by PFAS sample solution. All NMR sample solutions were thoroughly mixed using a vortex mixer before transferring them to Wilmad NMR tubes (600 MHz) with 5 mm diameter and 7-inch length. Exact composition of each NMR sample is given in **Table S1**. Bruker 600 MHz NMR instrument was used for data collection, and the configuration of the spectrometer is given in **Table S2**.

MestReNova software was used for NMR spectral analysis. Automatic baseline and phase correction were applied to the spectra. ^{19}F peak of HFP was set at -164.90 parts per million (ppm).[4] Integration of all NMR peaks was performed manually. The integral of the terminal CF_3 group of PFAS molecules was used for quantification while integral of DFP NMR peak was used as reference. Integrations of the NMR peaks were converted into molar concentrations using following equation,

$$C_{PFAS} = \frac{C_{DFP} n_{DFP} I_{PFAS}}{I_{DFP} n_{PFAS}} \times \frac{V_T}{V_{PFAS}} \tag{S1}$$

where C_{PFAS} is the concentration of PFAS (mM), C_{DFP} is the concentration of DFP (mM), n_{DFP} is the number of fluorine atoms in DFP, I_{PFAS} is the integration of CF_3 peak of PFAS molecule, I_{DFP}

is the integration of NMR peak of DFP, n_{PFAS} is the number of fluorine atoms in terminal group of PFAS ($-CF_3$), V_T is the total volume of NMR sample, and V_{PFAS} is the volume of PFAS solution used to make NMR sample solution.

Table S1. Composition of the samples prepared for ^{19}F-NMR spectroscopy. The solutions were prepared in 2 mL glass vials before transferring them to NMR tubes.

Component	Concentration	Volume (µL)
D_2O	-	55
Hexafluorobenzene (standard for chemical shift)	5% (v/v) in IPA	10
2,6-difluorophenol (standard for integration)	2.8 mM in DMSO	28
Cr(III) acetylacetonate (relaxation agent)	50.6 mM in MeOH	62
$PFAS_{(aq)}$ (TFA, PFBA, or PFOA)	-	472

Table S2. NMR spectroscopy parameters for all ^{19}F-NMR spectra. Bruker 600 MHz NMR instrument was configured to these settings for all quantitative ^{19}F-NMR spectral analyses.

Parameter	Value	Unit
Probe Temperature	298	K
Pulse Angle	90	degrees
Size of FD (TD)	131580	
Number of Dummy Scans (DS)	4	
Loop Count (TD0)	1	
Number of Scans (NS)	500	
Spectral Width (SW)	233	ppm
Acquisition Time (AQ)	0.5	s
FID Resolution (FIDRES)	2	Hz
Filter Width (FW)	2.4×10^8	Hz
Delay (D1)	2.5	s
Receiver Gain (RG)	101	
Dwell Time (DW)	3.8	µs

Intraparticle diffusion model (IPD)

The adsorption process on a porous adsorbent can have three steps: external diffusion (adsorbate diffuses to the external surface of the adsorbent), intraparticle diffusion (IPD, adsorbate diffuses through the adsorbent pores to reach the sorption site), and final sorption of the adsorbate. The last of these steps is usually fast and has a negligible effect on the sorption kinetics. Since the pseudo-second order (PSO) kinetic model does not describe the mechanism of the sorption process, another model, the intraparticle diffusion model can give some insights.[5] The IPD model equation is expressed as

$$q_t = \frac{6q_e}{r}\left(\frac{D}{\pi}\right)^{0.5} t^{0.5} = k_d t^{0.5} + C \tag{S2}$$

where D (mm^2 h^{-1}) is intraparticle diffusivity, r (mm) is the radius of the particle, k_d (mmol g^{-1} $h^{-0.5}$) is the intraparticle diffusion rate constant, and C (mmol g^{-1}) is the boundary layer thickness coefficient. The data q_t is plotted versus $t^{0.5}$, and linear fitting is performed. When the fit passes through origin, the IPD model assumes that external diffusion is negligible and intraparticle diffusion is the only rate-controlling step in homogeneous solutions. However, positive C value is consistent with a boundary layer formation that also controls the adsorbent diffusion on the adsorbate and pore accessibility.

2. Supplemental Results

Polymers syntheses

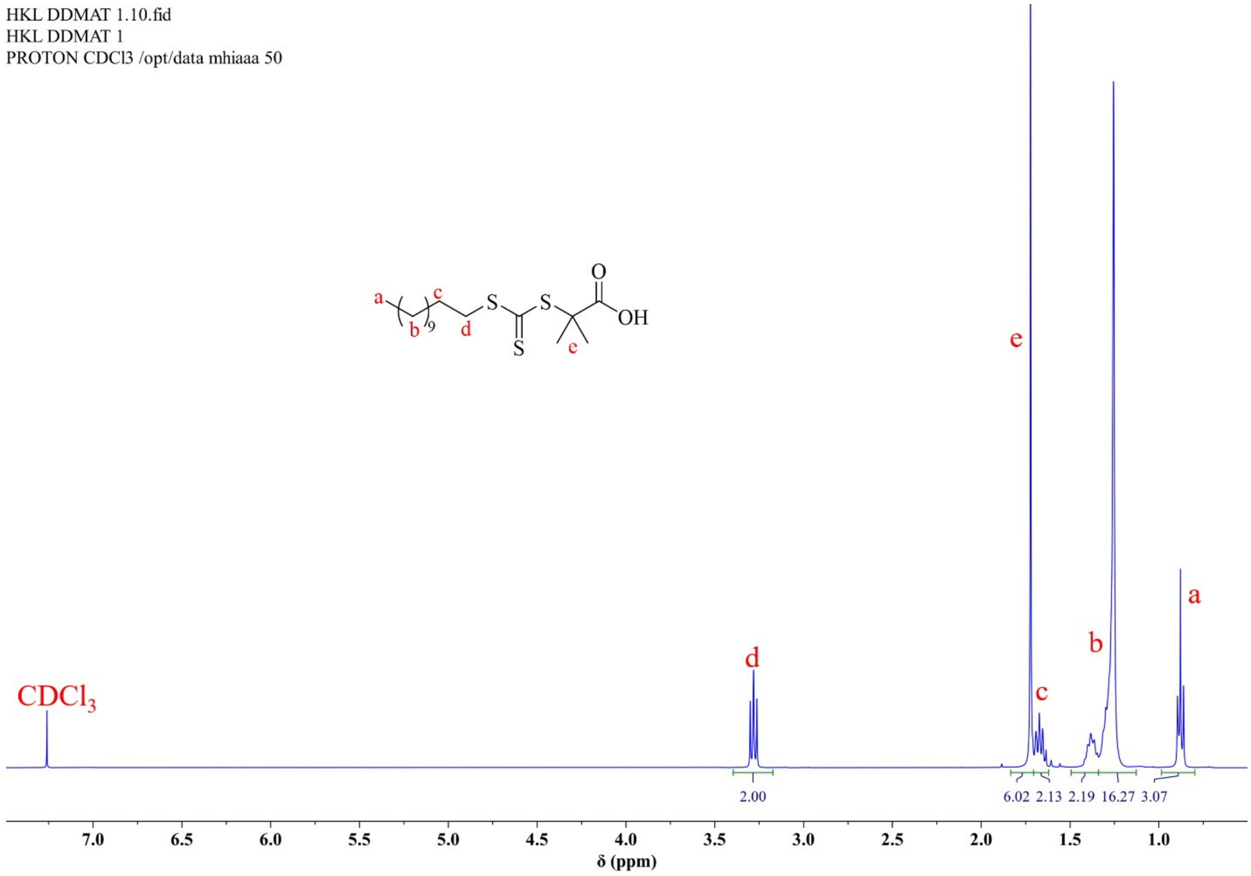

Figure S1. The ^{1}H-NMR spectrum of DDMAT in $CDCl_3$ with all the peaks labelled and integrated.

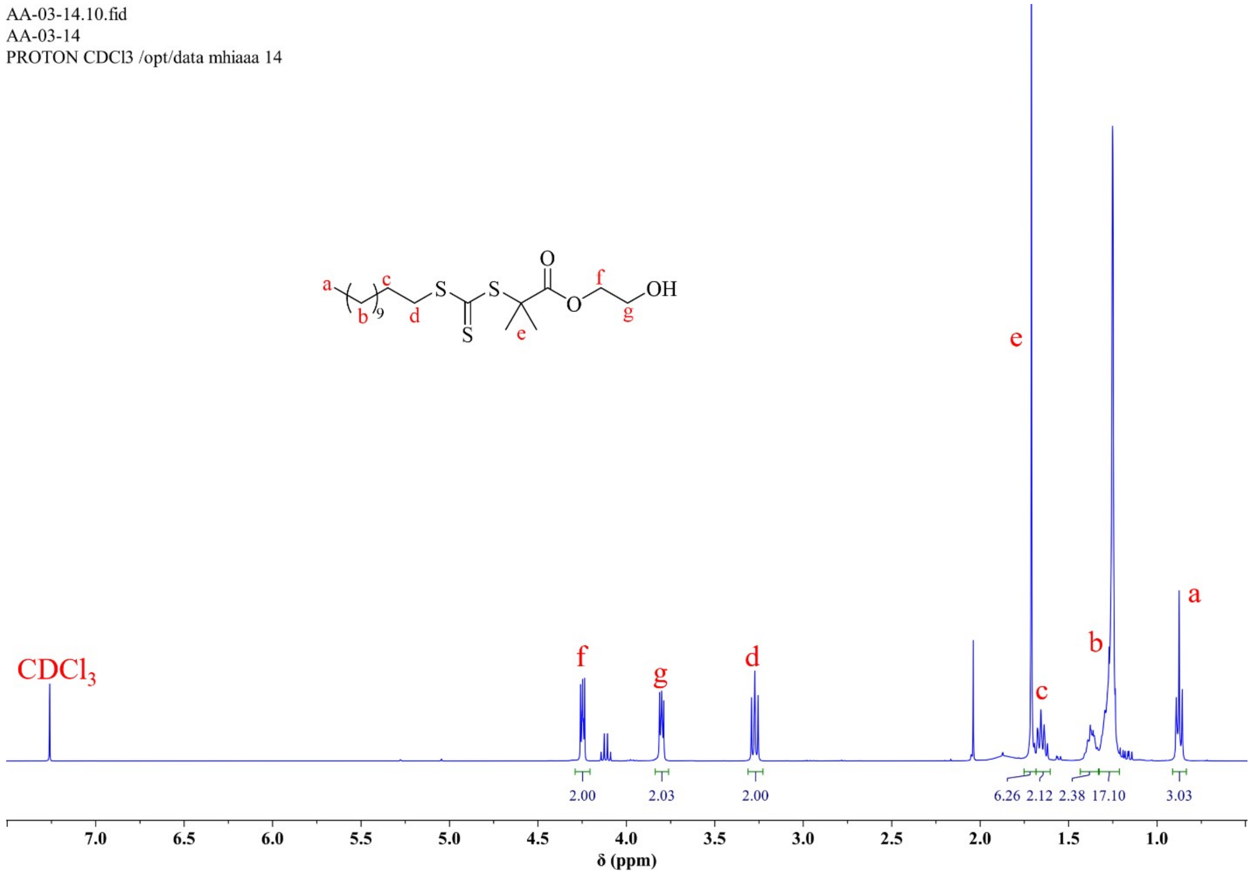

Figure S2. The ^{1}H-NMR spectrum of DDMAT-OH in $CDCl_3$ with all the peaks labelled and integrated.

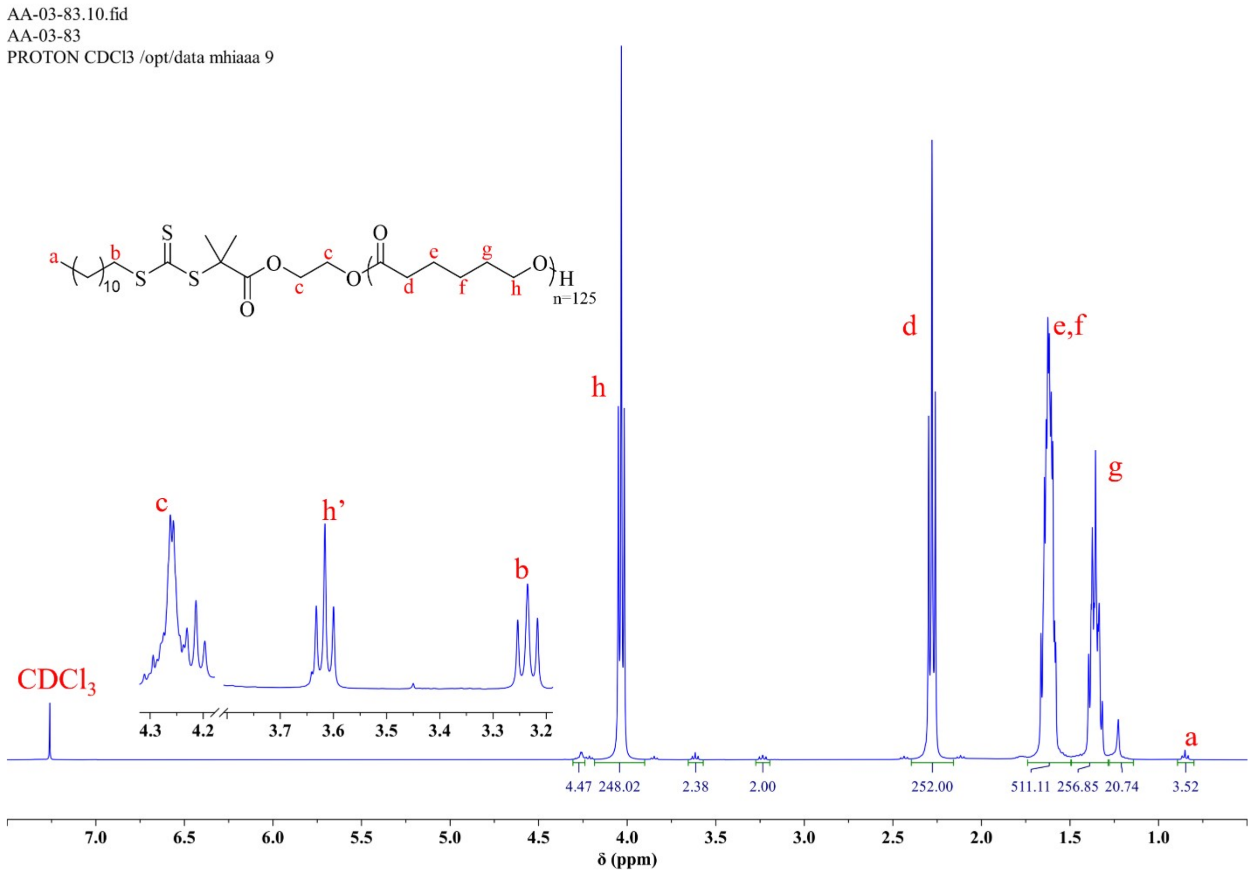

Figure S3. The ^{1}H-NMR spectrum of PCL-CTA in $CDCl_3$ with all the peaks labelled and integrated.

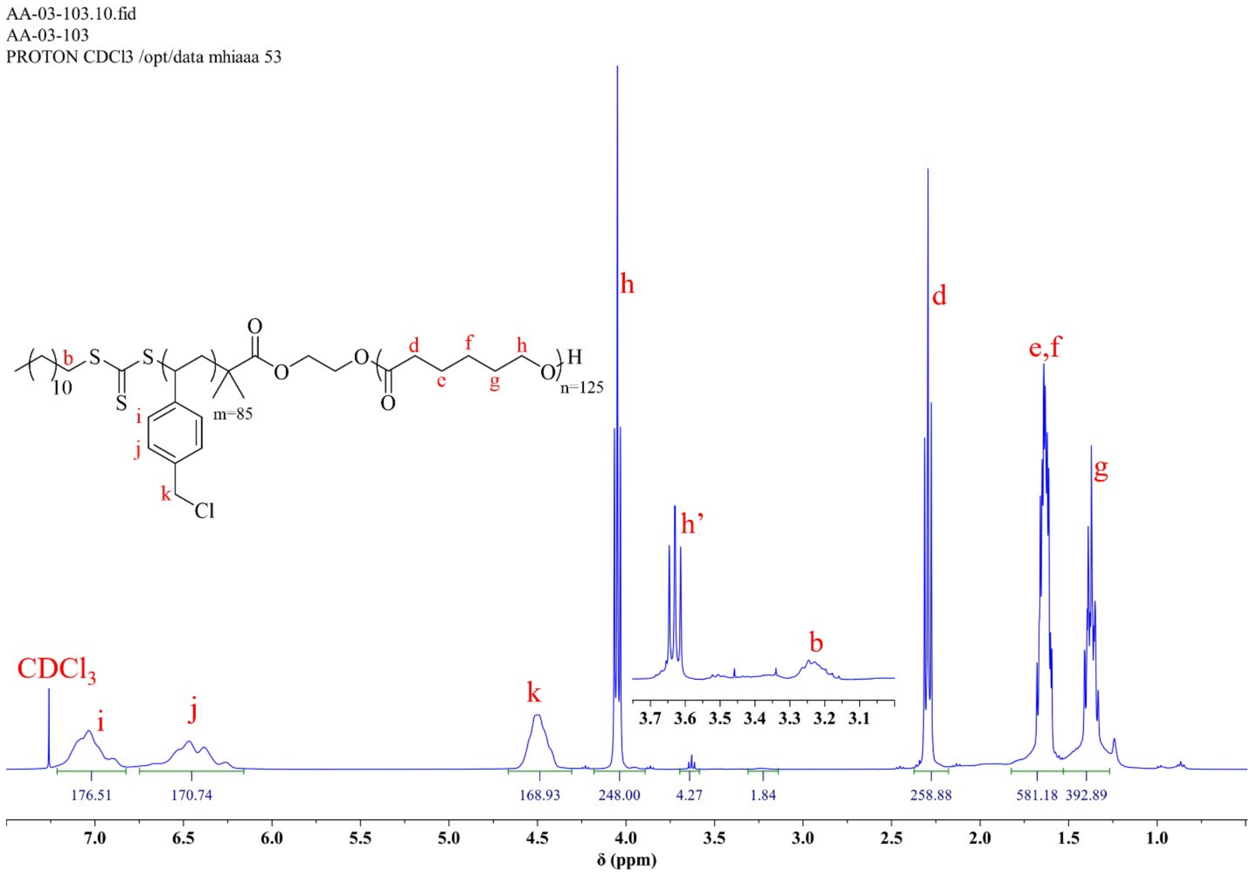

Figure S4. The ^{1}H-NMR spectrum of PCL-*b*-PVBC-CTA in $CDCl_3$ with all the peaks labelled and integrated.

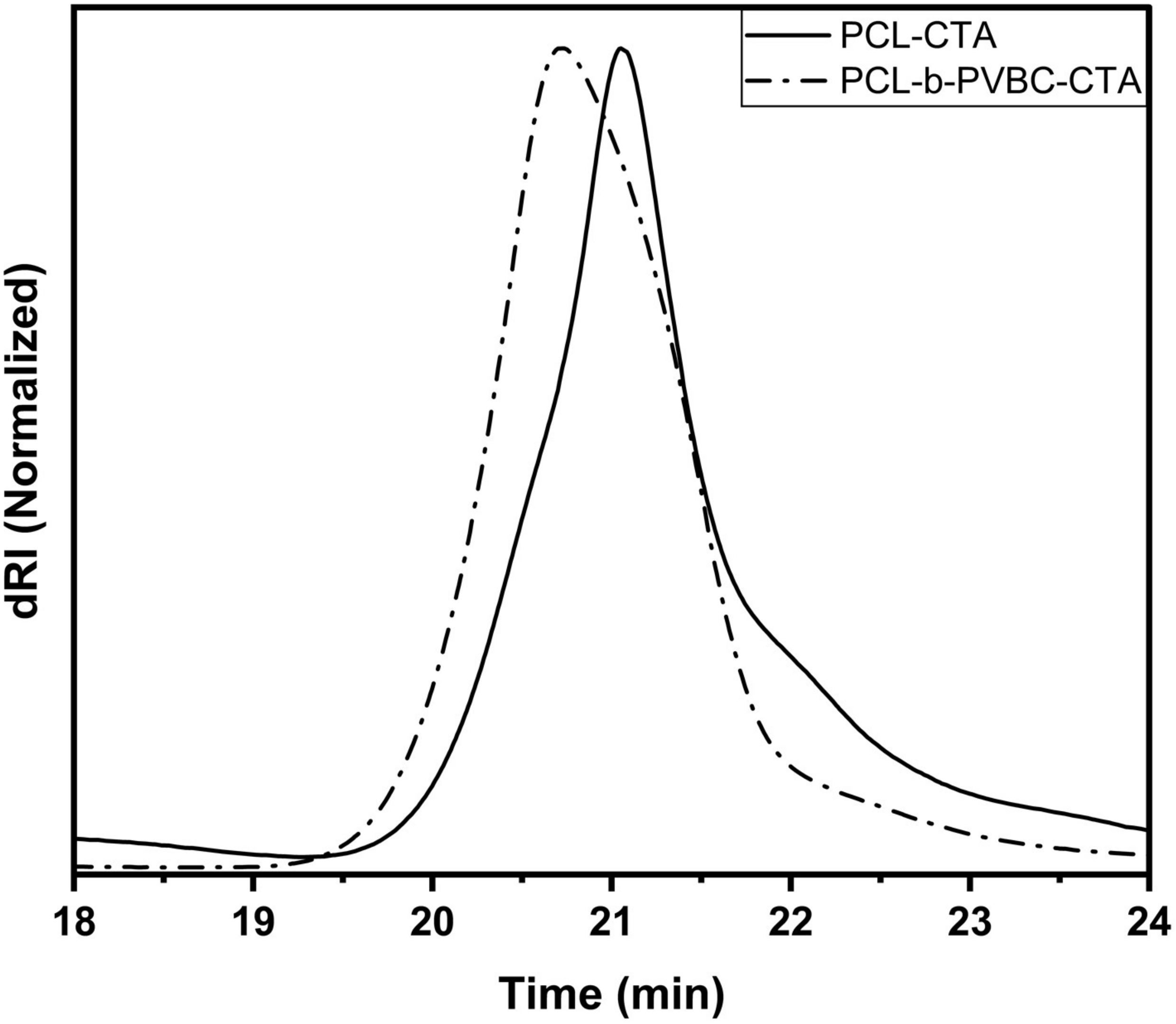

Figure S5. THF-SEC dRI (normalized) traces of PCL-CTA and PCL-*b*-PVBC-CTA. After adding a PVBC block to the PCL homopolymer, the SEC trace shifted to a lower elution time (high molecular weight).

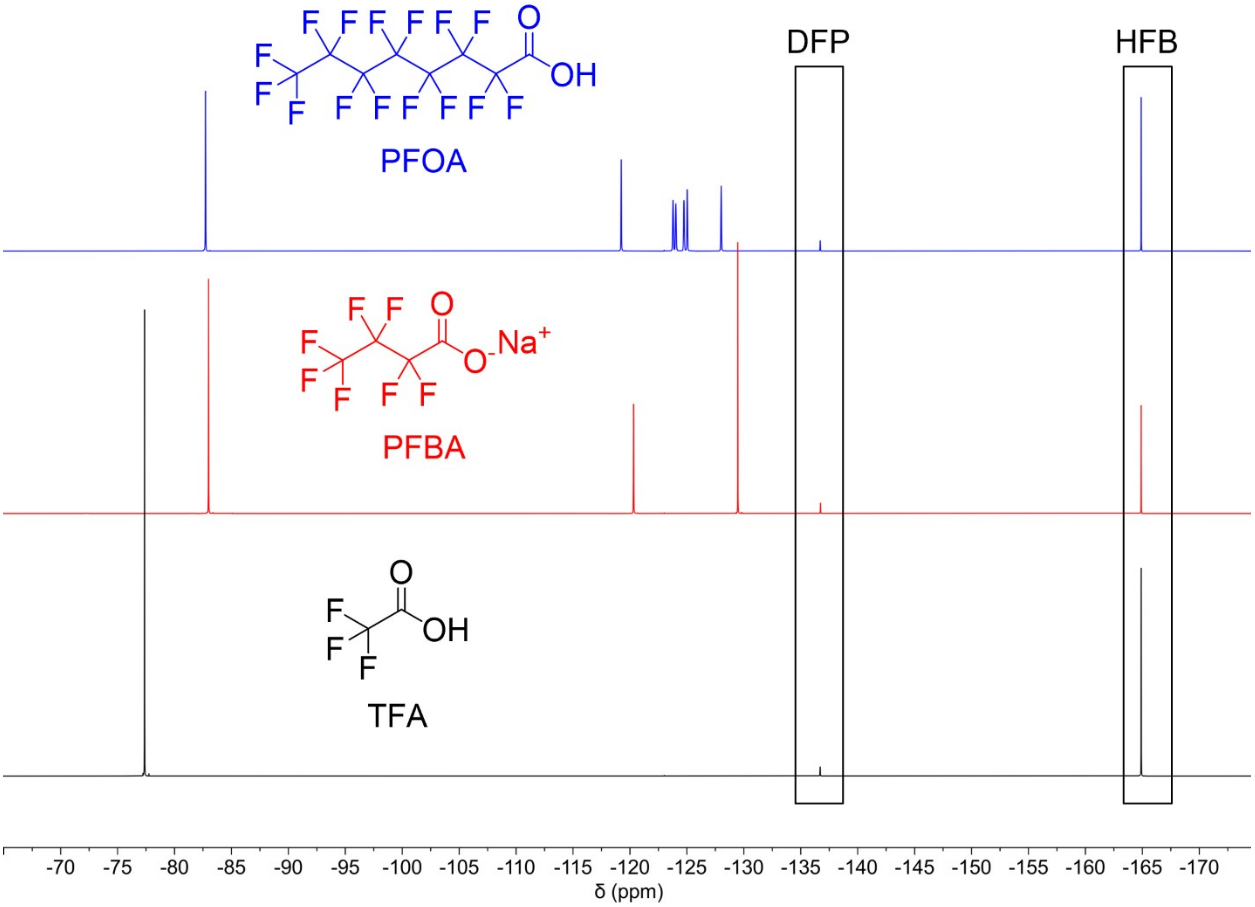

Figure S6. ^{19}F-NMR spectra of TFA, PFBA, and PFOA with DFP and HFB as internal standards. Integrals of the characteristic $–CF_3$ peaks (–76 ppm to –83 ppm versus $–CCl_3F$) were used for quantitative analysis.

Bead characterization

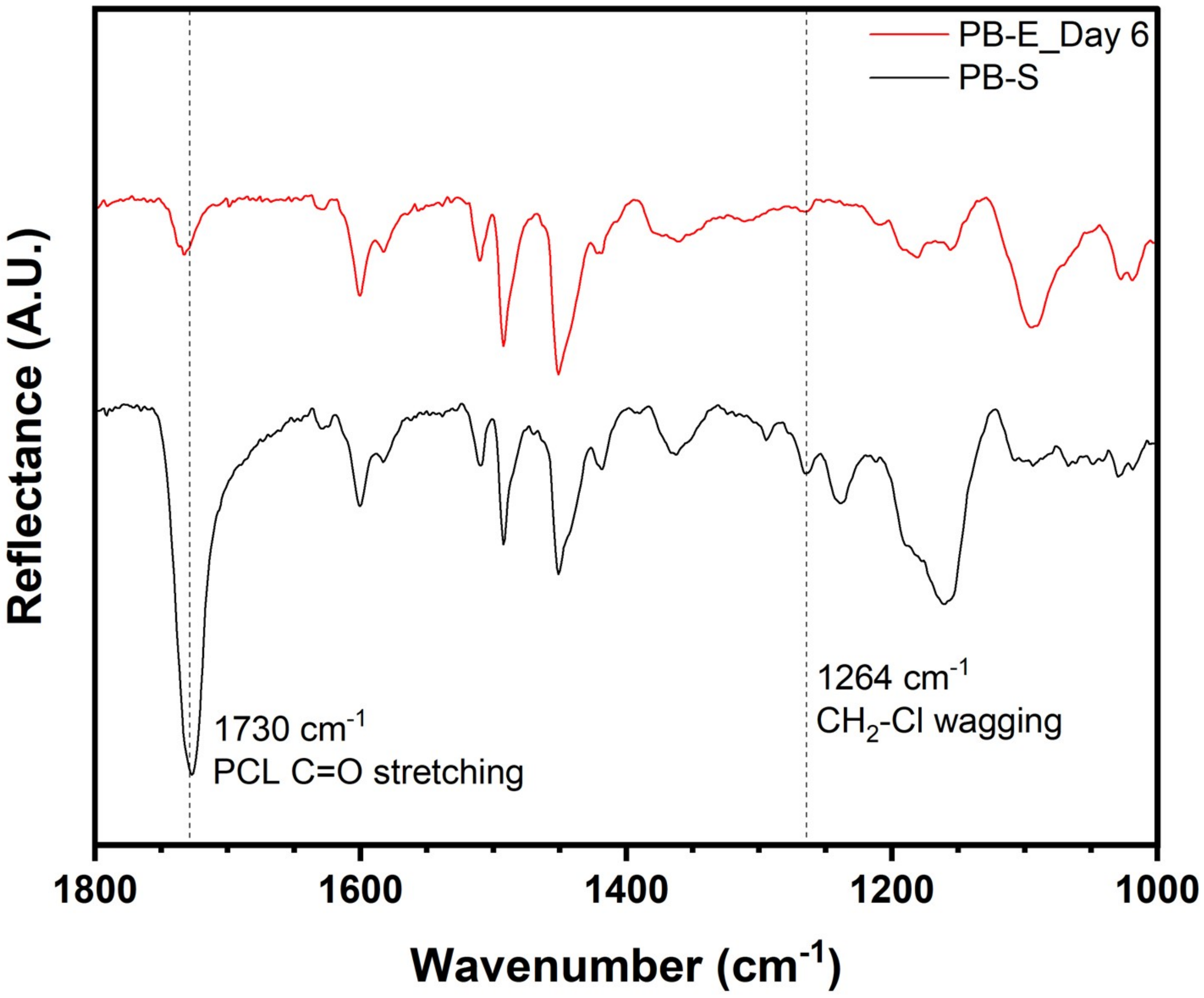

Figure S7. ATR-FTIR spectra of PB-S (black) and alkaline-etched beads after 6 days (red). The 1730 cm^{-1} peak corresponds to C=O stretching in PCL: the 1264 cm^{-1} peak results from CH_2-Cl wagging in PVBC.

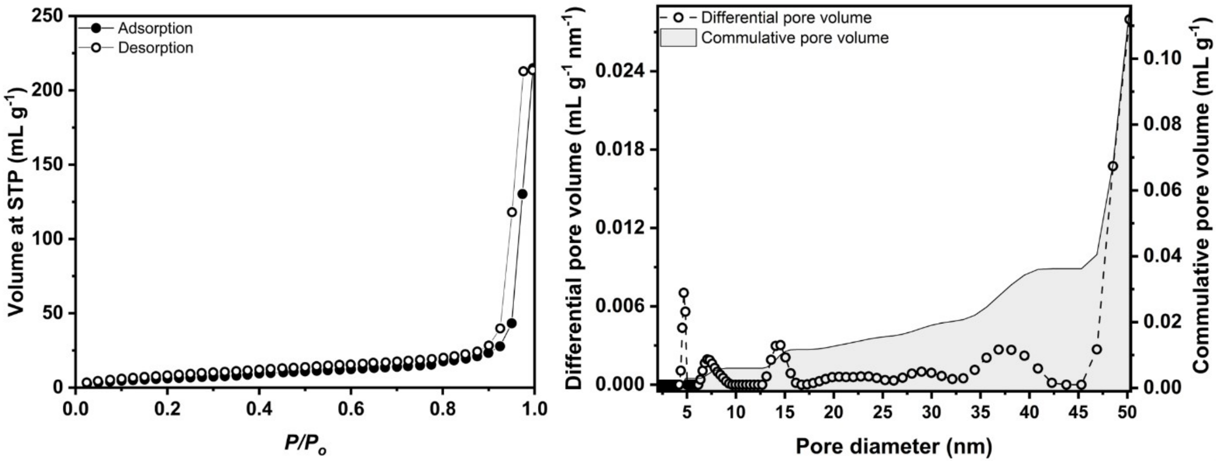

Figure S8. N_2 sorption data for IRA 900. (a) Nitrogen sorption isotherms with filled symbols for adsorption and empty symbols for desorption. (b) Pore size distribution using the QSDFT model using the adsorption branch of the isotherm and assuming slit/cylindrical pores on the carbon surface. The mode diameter of the pores was 50 nm, with 0.12 mL g^{-1} pore volume and 25 m^2 g^{-1} surface, as obtained with the BET model.

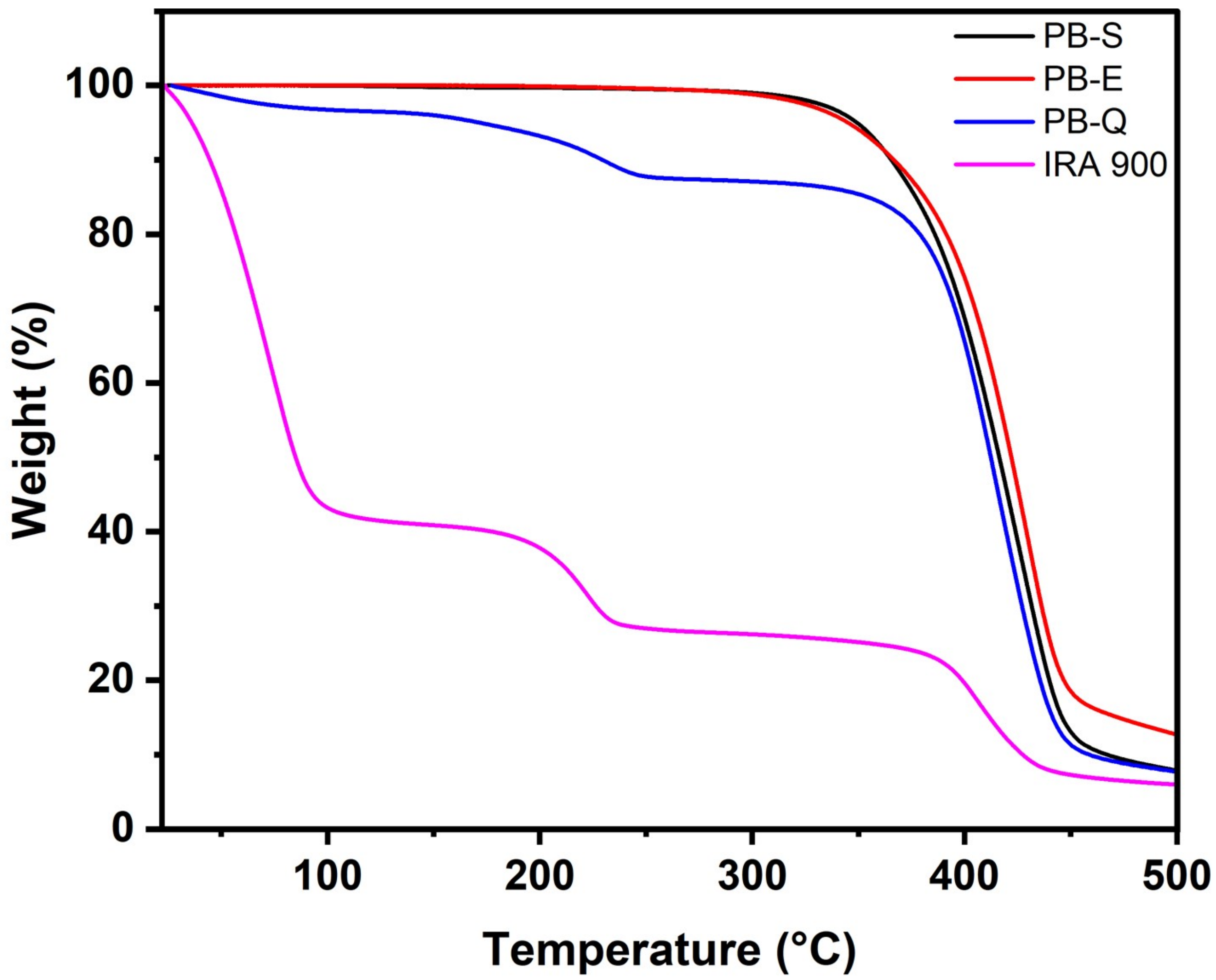

Figure S9. TGA traces of PB-S (black) and PB-E (red), PB-Q (blue), and IRA 900 (magenta). PB-S and PB-E were dry and stable below 300 °C. PB-Q and IRA 900 comprised 5% and 57% water, respectively, which evaporated at around 100 °C. The mass loss after 150 °C is attributed to the loss of quaternary ammonium groups in the quaternized beads.

Ion exchange capacity (IEC) measurements

IEC of the beads was measured using conductometric and pH titration methods. With both methods, counter ions (Cl^-) were quantified, indirectly giving the number of quaternary ammonium ions ($-N^+(CH_3)_3$) on PB-Q. In conductometric titration, the beads were sonicated in $NaNO_{3(aq)}$ solution to exchange all $Cl^–$ ions in the beads with $NO_3^–$ ions. Then, the $NaNO_{3(aq)}$ solution was titrated against the $AgNO_{3(aq)}$ solution, and the conductivity of the solution was monitored. In the early stage of conductometric titration, adding $AgNO_{3(aq)}$ results in the reaction between Ag^+ and $Cl^–$ ions, forming $AgCl_{(s)}$. This results in a negligible change in conductivity. However, the conductivity of the solution starts increasing linearly once there is an excess of Ag^+ with respect to $Cl^–$ ions. The volume of $AgNO_{3(aq)}$ used to reach the onset point of this increase in conductivity is used to calculate the amount of $Cl^–$ ions initially present in the beads and, therefore, the number of quaternary ammonium groups (positive charges).

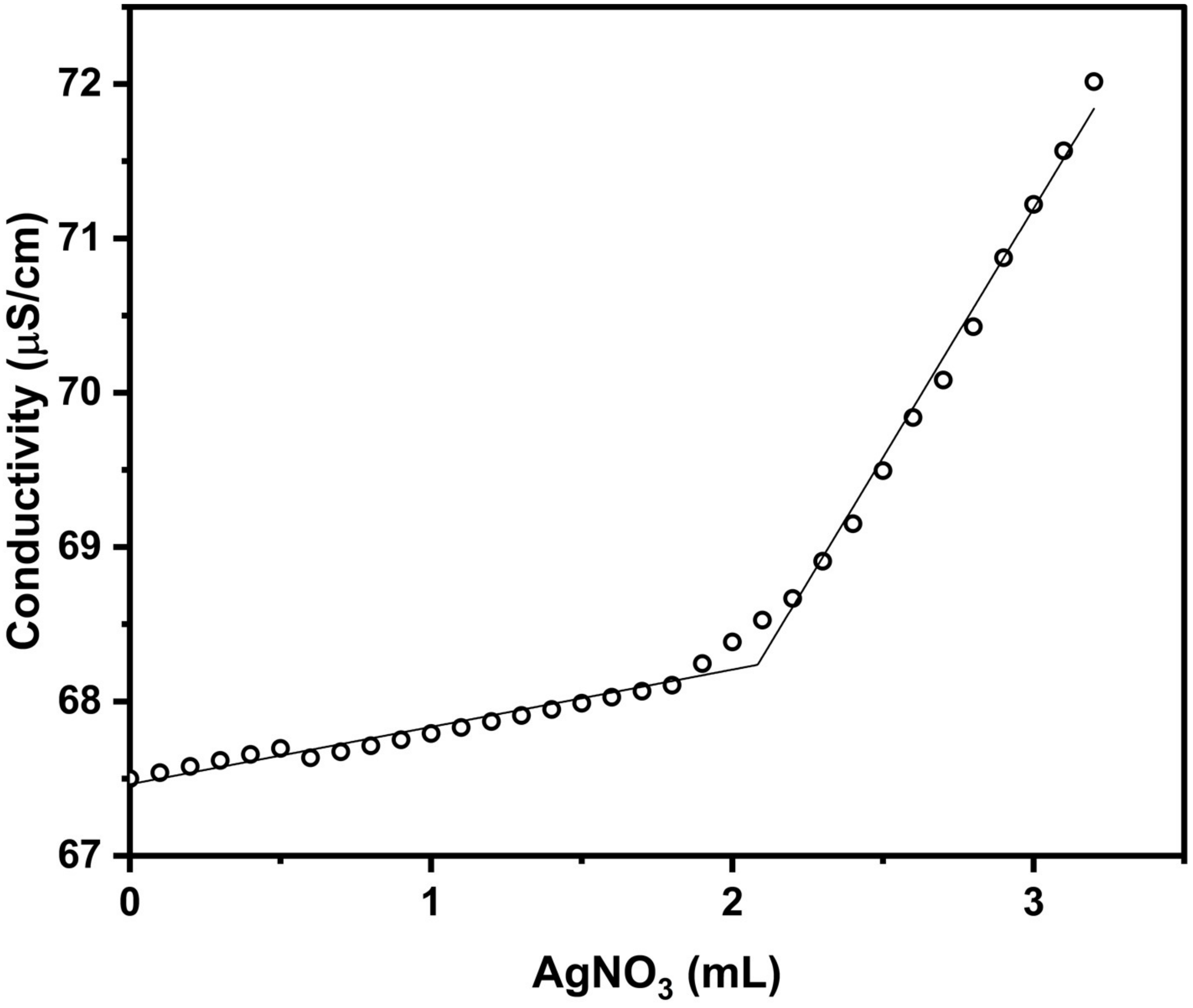

Figure S10. Conductometric titration graph for ion exchange capacity (IEC) measurement of PB-Q. The conductivity of the bead suspension in the deionized water did not increase until all the Cl^- ions had reacted with Ag^+.

In pH titration, Cl^- ions were first exchanged with OH^- ions by treating the beads with $NaOH_{(aq)}$ solution. In the second step, OH^- ions were exchanged with Cl^- ions by treating the beads with $NaCl_{(aq)}$ solution. This NaCl solution was titrated against an $HCl_{(aq)}$ solution, and the change in the pH was monitored.

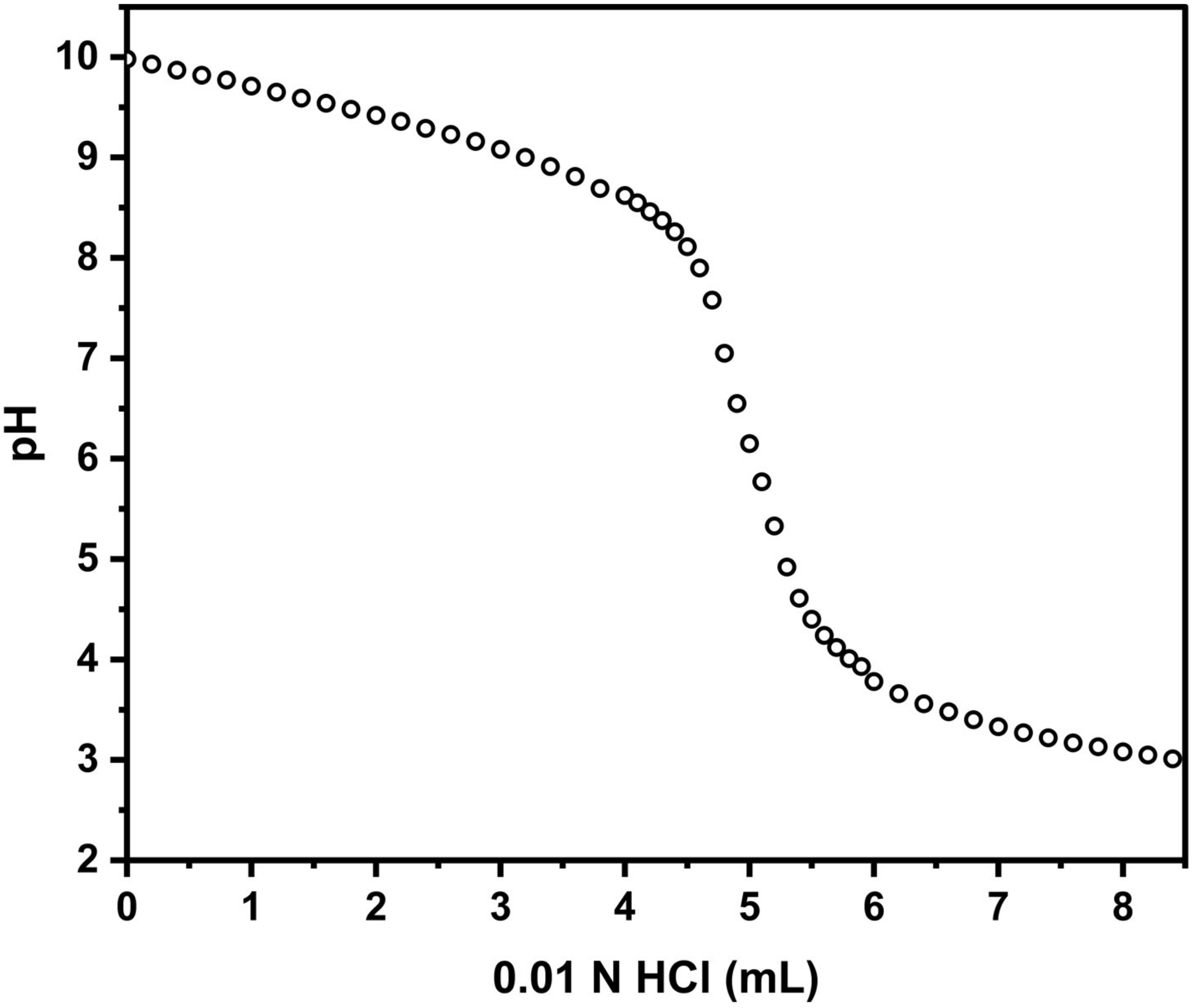

Figure S11. pH titration graph for IEC measurement of PB-Q. The exchange of Cl^- counter ions with OH^- ions (using $NaOH_{(aq)}$) followed by another exchange of OH^- with Cl^- (using NaCl) gave a basic $NaCl_{(aq)}$ solution. The OH^- ions in the $NaCl_{(aq)}$ solution were titrated against 0.01 N $HCl_{(aq)}$.

Kinetic data and fitting

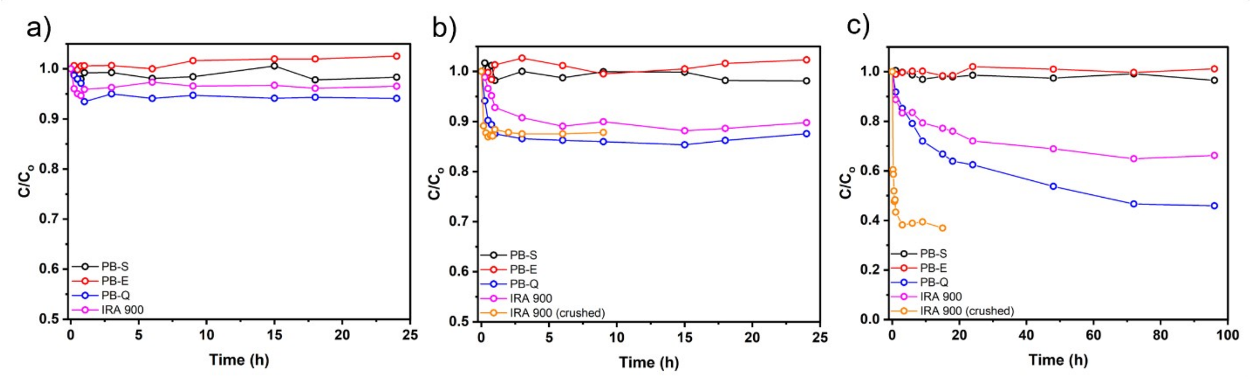

Figure S12. Sorption of PFAS vs time using PB-S (black), PB-E (red), and PB-Q (blue), as well as IRA 900 (magenta). a) TFA, b) PFBA, and c) PFOA. For all data points, 10 mg wet beads were added to 16 mL of 2 mM PFAS (TFA, PFBA, or PFOA) solution in a glass vial, and vials were placed on an orbital shaker at 140 rpm at room temperature.

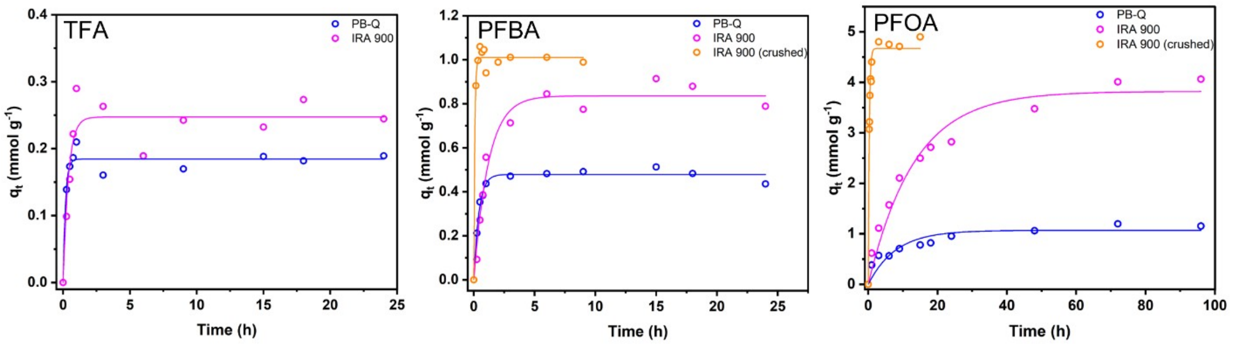

Figure S13. Sorption kinetics of PFAS (TFA, PFBA, or PFOA) with 10 mg (wet mass) PB-Q, IRA900 and IRA 900 (crushed) separately in 16 mL of 2 mM PFAS solution for each data point. Values for q_t were corrected to the dry mass of the adsorbent. Averages of the duplicates were plotted. Solid lines represent pseudo-firs order (PFO) kinetic model fitting.

Table S3. Kinetic parameters of the PFO model for the sorption of TFA, PFBA, and PFOA to PB-Q, IRA 900 and IRA 900 (crushed).

Adsorbent	Adsorbate	Pseudo-first order parameters		
		q_e (mmol g^{-1})	k_1 (h^{-1})	R^2
PB-Q	TFA	0.180 ± 0.005	5.8 ± 1.2	0.95
	PFBA	0.480 ± 0.008	2.4 ± 0.2	0.98
	PFOA	1.10 ± 0.07	0.130 ± 0.032	0.84
IRA 900 (whole)	TFA	0.250 ± 0.013	2.60 ± 0.65	0.86
	PFBA	0.840 ± 0.025	0.84 ± 0.10	0.97
	PFOA	3.80 ± 0.162	0.076 ± 0.009	0.96
IRA 900 (crushed)	PFBA	1.00 ± 0.01	13.0 ± 1.7	0.99
	PFOA	4.70 ± 0.10	3.5 ± 0.3	0.98

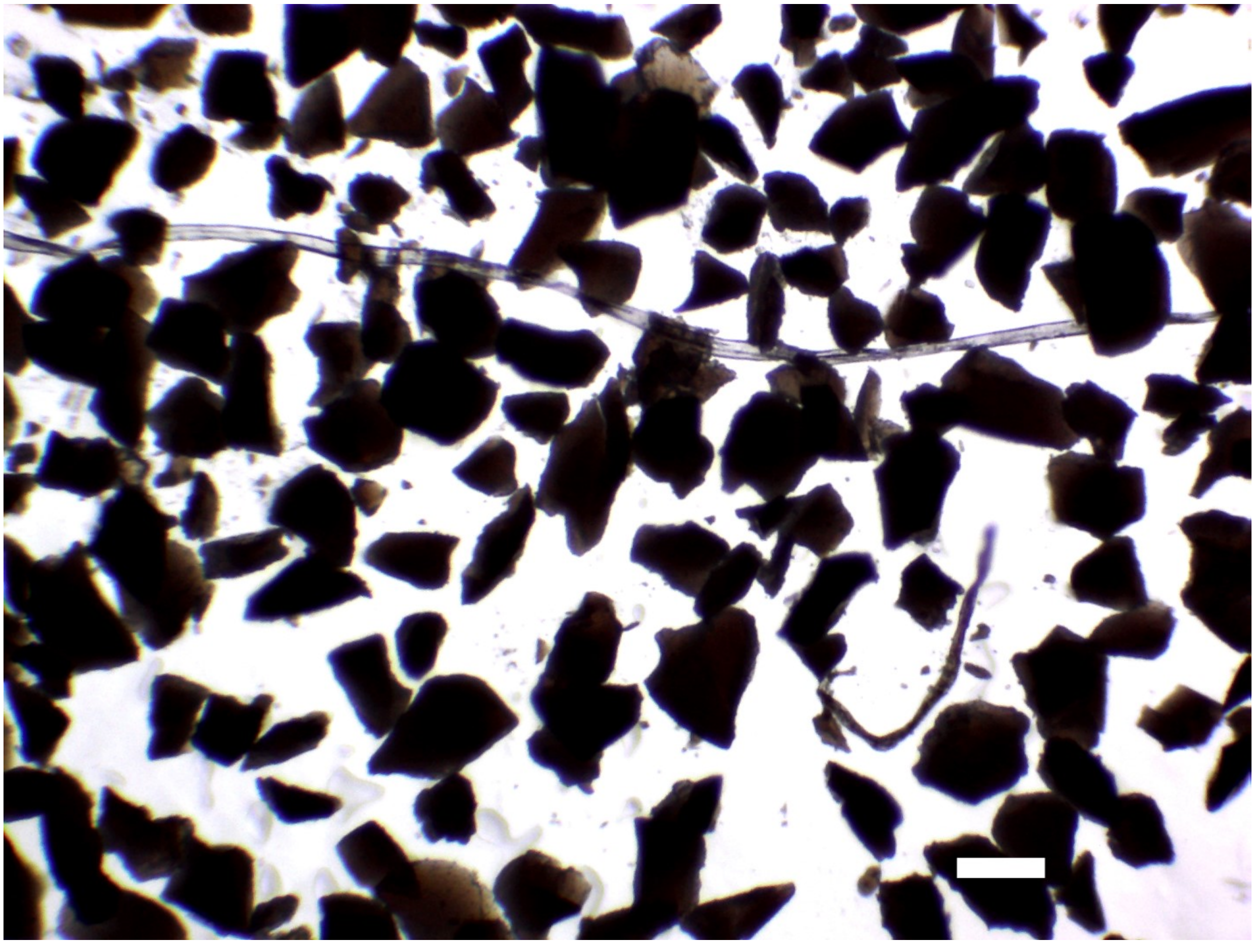

Figure S14. Digital microscopic image of IRA 900 (crushed) with scale bar representing 100 μm.

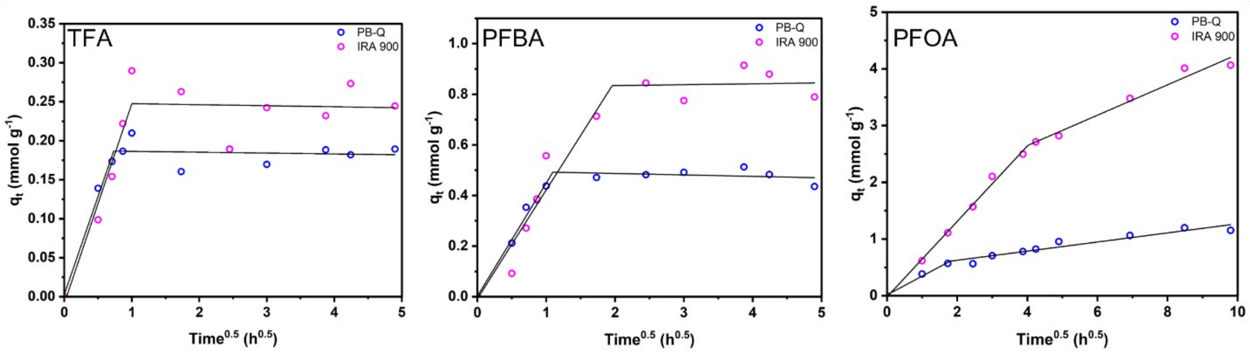

Figure S15. IPD model fitting PFAS sorption kinetics data from a batch sorption experiment.

Table S4. Freundlich isotherm parameters for sorption of TFA, PFBA, and PFOA on PB-Q and IRA 900.

Adsorbent	Adsorbate	Freundlich parameters		
		K_F (mmol$^{(1-1/n)}$ L$^{1/n}$ g^{-1})	n	R^2
PB-Q	TFA	0.150 ± 0.006	4.0 ± 0.8	0.80
	PFBA	0.370 ± 0.010	3.9 ± 0.4	0.95
	PFOA	1.30 ± 0.11	11.0 ± 4.2	0.83
IRA 900 (whole)	TFA	0.270 ± 0.008	1.3 ± 0.1	0.98
	PFBA	0.46 ± 0.03	1.10 ± 0.14	0.94
	PFOA	6.60 ± 0.87	2.70 ± 0.49	0.83

References

1 J. T. Lai, D. Filla and R. Shea, Functional Polymers from Novel Carboxyl-Terminated Trithiocarbonates as Highly Efficient RAFT Agents, *Macromolecules*, 2002, **35**, 6754–6756.

2 X. Dong, A. C. Obermeyer and B. D. Olsen, Three-Dimensional Ordered Antibody Arrays Through Self-Assembly of Antibody–Polymer Conjugates, *Angew. Chem. Int. Ed.*, 2017, **56**, 1273–1277.

3 K. A. Faber, W. C. K. Pomerantz, J. L. Gray, L. E. Hubbard, D. W. Kolpin and W. A. Arnold, Revealing Organofluorine Contamination in Effluents and Surface Waters with Complementary Analytical Approaches: Fluorine-19 Nuclear Magnetic Resonance Spectroscopy (^{19}F-NMR) and Liquid Chromatography-Tandem Mass Spectrometry (LC-MS/MS), *Environ. Sci. Technol.*, 2025, **59**, 14695–14706.

4 A. P. Bhat, T. F. Mundhenke, Q. T. Whiting, A. A. Peterson, W. C. K. Pomerantz and W. A. Arnold, Tracking Fluorine during Aqueous Photolysis and Advanced UV Treatment of Fluorinated Phenols and Pharmaceuticals Using a Combined ^{19}F-NMR, Chromatography, and Mass Spectrometry Approach, *ACS Environ. Au*, 2022, **2**, 242–252.

5 Q. Yu, R. Zhang, S. Deng, J. Huang and G. Yu, Sorption of perfluorooctane sulfonate and perfluorooctanoate on activated carbons and resin: Kinetic and isotherm study, *Water Res.*, 2009, **43**, 1150–1158.
